# *SCN10A-short* gene therapy to restore conduction and protect against malignant cardiac arrhythmias

**DOI:** 10.1093/eurheartj/ehaf053

**Published:** 2025-02-20

**Authors:** Jianan Wang, Arie O Verkerk, Ronald Wilders, Yingnan Zhang, Kelly Zhang, Adityo Prakosa, Mathilde R Rivaud, E Madelief J Marsman, Arie R Boender, Mischa Klerk, Lianne Fokkert, Berend de Jonge, Klaus Neef, Osne F Kirzner, Connie R Bezzina, Carol Ann Remme, Hanno L Tan, Bastiaan J Boukens, Harsha D Devalla, Natalia A Trayanova, Vincent M Christoffels, Phil Barnett, Gerard J J Boink

**Affiliations:** Department of Medical Biology, Amsterdam Cardiovascular Sciences, Amsterdam University Medical Centers, University of Amsterdam, Meibergdreef 15, Amsterdam 1105 AZ, The Netherlands; Department of Medical Biology, Amsterdam Cardiovascular Sciences, Amsterdam University Medical Centers, University of Amsterdam, Meibergdreef 15, Amsterdam 1105 AZ, The Netherlands; Department of Clinical and Experimental Cardiology, Amsterdam Cardiovascular Sciences, Amsterdam University Medical Centers, University of Amsterdam, Meibergdreef 15, Amsterdam 1105 AZ, The Netherlands; Department of Medical Biology, Amsterdam Cardiovascular Sciences, Amsterdam University Medical Centers, University of Amsterdam, Meibergdreef 15, Amsterdam 1105 AZ, The Netherlands; Department of Biomedical Engineering, Johns Hopkins University, Baltimore, MD 21218, USA; Department of Biomedical Engineering, Johns Hopkins University, Baltimore, MD 21218, USA; Department of Biomedical Engineering, Johns Hopkins University, Baltimore, MD 21218, USA; Department of Medical Biology, Amsterdam Cardiovascular Sciences, Amsterdam University Medical Centers, University of Amsterdam, Meibergdreef 15, Amsterdam 1105 AZ, The Netherlands; Department of Clinical and Experimental Cardiology, Amsterdam Cardiovascular Sciences, Amsterdam University Medical Centers, University of Amsterdam, Meibergdreef 15, Amsterdam 1105 AZ, The Netherlands; Department of Medical Biology, Amsterdam Cardiovascular Sciences, Amsterdam University Medical Centers, University of Amsterdam, Meibergdreef 15, Amsterdam 1105 AZ, The Netherlands; PacingCure B.V., Roetersstraat 35, Amsterdam 1018 WB, The Netherlands; Department of Medical Biology, Amsterdam Cardiovascular Sciences, Amsterdam University Medical Centers, University of Amsterdam, Meibergdreef 15, Amsterdam 1105 AZ, The Netherlands; Department of Medical Biology, Amsterdam Cardiovascular Sciences, Amsterdam University Medical Centers, University of Amsterdam, Meibergdreef 15, Amsterdam 1105 AZ, The Netherlands; Department of Medical Biology, Amsterdam Cardiovascular Sciences, Amsterdam University Medical Centers, University of Amsterdam, Meibergdreef 15, Amsterdam 1105 AZ, The Netherlands; PacingCure B.V., Roetersstraat 35, Amsterdam 1018 WB, The Netherlands; Netherlands Heart Institute, Moreelsepark 1, Utrecht 3511 EP, The Netherlands; PacingCure B.V., Roetersstraat 35, Amsterdam 1018 WB, The Netherlands; Department of Anaesthesiology, Amsterdam University Medical Centers, De Boelelaan 1117, Amsterdam 1081 HV, The Netherlands; Department of Clinical and Experimental Cardiology, Amsterdam Cardiovascular Sciences, Amsterdam University Medical Centers, University of Amsterdam, Meibergdreef 15, Amsterdam 1105 AZ, The Netherlands; Department of Clinical and Experimental Cardiology, Amsterdam Cardiovascular Sciences, Amsterdam University Medical Centers, University of Amsterdam, Meibergdreef 15, Amsterdam 1105 AZ, The Netherlands; Department of Clinical and Experimental Cardiology, Amsterdam Cardiovascular Sciences, Amsterdam University Medical Centers, University of Amsterdam, Meibergdreef 15, Amsterdam 1105 AZ, The Netherlands; PacingCure B.V., Roetersstraat 35, Amsterdam 1018 WB, The Netherlands; Netherlands Heart Institute, Moreelsepark 1, Utrecht 3511 EP, The Netherlands; Department of Medical Biology, Amsterdam Cardiovascular Sciences, Amsterdam University Medical Centers, University of Amsterdam, Meibergdreef 15, Amsterdam 1105 AZ, The Netherlands; Department of Physiology, Cardiovascular Research Institute Maastricht (CARIM), Maastricht University, Universiteitssingel 50, Maastricht 6229 ER, The Netherlands; Department of Medical Biology, Amsterdam Cardiovascular Sciences, Amsterdam University Medical Centers, University of Amsterdam, Meibergdreef 15, Amsterdam 1105 AZ, The Netherlands; Department of Biomedical Engineering, Johns Hopkins University, Baltimore, MD 21218, USA; Alliance for Cardiovascular Diagnostic and Treatment Innovation, Johns Hopkins University, Baltimore, MD 21218, USA; Department of Medical Biology, Amsterdam Cardiovascular Sciences, Amsterdam University Medical Centers, University of Amsterdam, Meibergdreef 15, Amsterdam 1105 AZ, The Netherlands; Department of Medical Biology, Amsterdam Cardiovascular Sciences, Amsterdam University Medical Centers, University of Amsterdam, Meibergdreef 15, Amsterdam 1105 AZ, The Netherlands; Department of Medical Biology, Amsterdam Cardiovascular Sciences, Amsterdam University Medical Centers, University of Amsterdam, Meibergdreef 15, Amsterdam 1105 AZ, The Netherlands; PacingCure B.V., Roetersstraat 35, Amsterdam 1018 WB, The Netherlands; Department of Cardiology, Amsterdam Cardiovascular Sciences, Amsterdam University Medical Centers, University of Amsterdam, Meibergdreef 9, Amsterdam 1105 AZ, The Netherlands

**Keywords:** Gene therapy, Cardiac arrhythmia, Sodium current, SCN5A, SCN10A, AAV

## Abstract

**Background and Aims:**

Life-threatening arrhythmias are a well-established consequence of reduced cardiac sodium current (*I*_Na_). Gene therapy approaches to increase *I*_Na_ have demonstrated potential benefits to prevent arrhythmias. However, the development of such therapies is hampered by the large size of sodium channels. In this study, *SCN10A-short* (*S10s*), a short transcript encoding the carboxy-terminal domain of the human neuronal sodium channel, was evaluated as a gene therapy target to increase *I*_Na_ and prevent arrhythmias.

**Methods:**

Adeno-associated viral vector overexpressing *S10s* was injected into wild type and *Scn5a*-haploinsufficient mice on which patch-clamp studies, optical mapping, electrocardiogram analyses, and ischaemia reperfusion were performed. *In vitro* and *in silico* studies were conducted to further explore the effect of *S10s* gene therapy in the context of human hearts.

**Results:**

Cardiac *S10s* overexpression increased cellular *I*_Na_, maximal action potential upstroke velocity, and action potential amplitude in *Scn5a*-haploinsufficient cardiomyocytes. *S10s* gene therapy rescues conduction slowing in *Scn5a*-haploinsufficient mice and prevented ventricular tachycardia induced by ischaemia-reperfusion in wild type mice. *S10s* overexpression increased maximal action potential upstroke velocity in human inducible pluripotent stem cell-derived cardiomyocytes and prevented inducible arrhythmias in simulated human heart models.

**Conclusions:**

*S10s* gene therapy may be effective to treat cardiac conduction abnormalities and associated arrhythmias.

## Structured Graphical Abstract

**Structured Graphical abstract:**
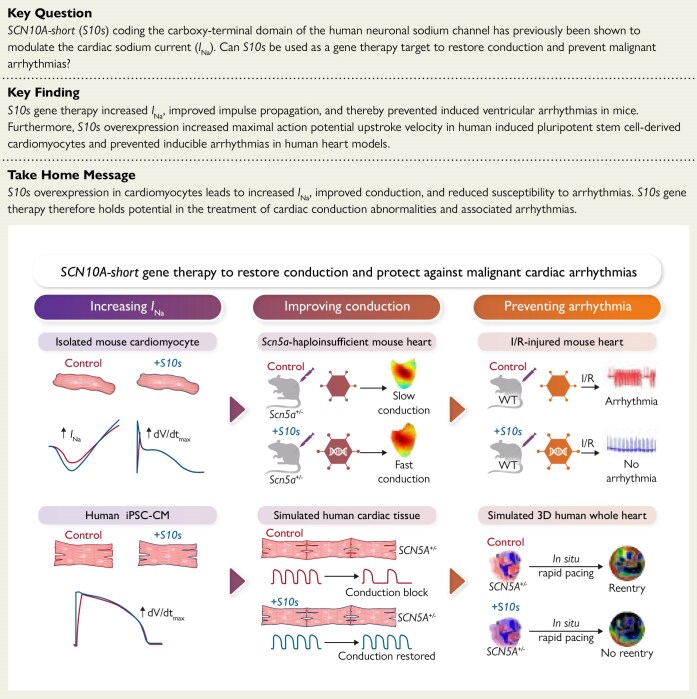
*SCN10A-short* (*S10s*) overexpression in cardiomyocytes increases sodium current (*I*_Na_) density and maximal action potential upstroke velocity (d*V*/d*t*_max_), thereby improving cardiac conduction and preventing inducible arrhythmias. Left panel: Cardiomyocytes isolated from mice injected with *S10s* showed increased *I*_Na_-driven d*V*/d*t*_max_ and increased *I*_Na_ density compared to control. Human induced pluripotent stem cell-derived cardiomyocyte (iPSC-CMs) transduced with *S10s* also showed increased d*V*/d*t*_max_ compared to control. Middle panel: *S10s* gene therapy increased ventricular conduction velocity in *Scn5a*-haploinsufficient mice. *S10s* gene therapy prevented conduction block in a simulated *SCN5A*-haploinsufficient human cardiac tissue model. Right panel: *S10s* gene therapy protected against ventricular arrhythmias in an ischaemia-reperfusion (I/R) mouse model and in a simulated human *SCN5A*-haploinsufficient whole-heart model. 3D, three-dimensional; WT, wild type.

**See the editorial comment for this article ‘Gene therapy targeting *I*_Na_ to treat life-threatening arrhythmias: beyond proof-of-concept?’, by P. Lugenbiel, https://doi.org10.1093/eurheartj/ehae930.**

**Translational perspective**

Cardiac sodium current (*I*_Na_) reduction is implicated in various arrhythmias including Brugada syndrome and ventricular tachycardia or fibrillation. Overexpression of *SCN10A-short* (*S10s*), a short transcript encoding the carboxy-terminal domain of the human neuronal sodium channel, leads to increased *I*_Na_ and increased maximal action potential upstroke velocity in both healthy and *Scn5a*-haploinsufficent cardiomyocytes. Gene therapy based on *S10s* expression improves cardiac conduction and prevents cardiac arrhythmias in both mouse and simulated human heart models. These results suggest that *S10s* gene therapy has the potential to be broadly applicable for the treatment of cardiac arrhythmias.

## Introduction

Ion channels play an important role in all aspects of heart function including rhythmicity and contractility.^1^ The *SCN5A*-encoded α-subunit of the cardiac sodium channel (Na_V_1.5) largely determines the cardiac sodium current (*I*_Na_), which is responsible for the initiation and propagation of the cardiac electrical excitation wavefront. A reduction of *I*_Na_ impairs the action potential (AP) upstroke and cardiac conduction, and is implicated in both acquired and inherited arrhythmia syndromes such as Brugada syndrome (BrS), progressive cardiac conduction disease (PCCD), sick sinus syndrome (SSS), atrial fibrillation (AF), and ventricular tachycardia or fibrillation (VT/VF).^2–5^

In recent years, gene therapy has emerged as a powerful tool to restore the function of damaged or dysfunctional cells and tissues.^6–8^ Restoration of cardiac *I*_Na_ by gene transfer of *SCN5A* represents a logical approach with the potential to provide a curative treatment. However, the large size of the *SCN5A* coding sequence is beyond the capacity of adeno-associated viral (AAV) vectors, complicating its clinical applicability in gene therapy. In 2021, Doisne *et al*. reported efficient expression of human *SCN5A* in neonatal mice utilizing a dual AAV-vector system.^9^ Yet the drawbacks of the dual AAV-vector system, such as its relatively low efficiency, the expression of unwanted products, and the need of two vector preparations, hamper clinical translation of this approach.^10^ Similarly, base editing has been successfully implemented to correct *SCN5A* mutations, but still requires the use of dual AAV-vector system due to the large size of the base editor,^11^ thus also harbouring the aforementioned drawbacks.

The challenges with dual AAV-vector system inspired the search for alternative strategies involving the expression of smaller transgenes. Overexpression of bacterial sodium channels effectively improved cardiac conduction *in vitro* and *in silico*.^12–14^ Yet the distinctly different gating properties could introduce pro-arrhythmic AP duration (APD) heterogeneity when expressed *in vivo*. Besides, anticipated higher immunogenicity poses important translational barriers. A different angle was taken in a recent study that demonstrated the potential value of the chaperone protein MOG1 as a means to suppress a BrS phenotype in Na_V_1.5*-*trafficking defect mouse models.^15^ While expression of MOG1 may represent a more translatable approach, the potential pleiotropic effects of MOG1 remain an important concern that could be difficult to address on an individual basis when considering eventual patient treatment.^16–18^ Moreover, MOG1 gene therapy was specifically designed to counter *SCN5A* trafficking mutations, which limits its application to a relatively small subset of genetic defects.

Recently, a naturally occurring cardiac-specific short transcript of *SCN10A* (*SCN10A-short*, here designated *S10s*) was discovered, which modulates the density of the Na_V_1.5-mediated *I*_Na_.^19^*S10s* is expressed in the sinus node, atria, and ventricular conduction system of the heart, and comprises the last seven exons of the neuronal sodium channel gene *SCN10A*. The predicted coding product of *S10s* contains the C-terminal portion of the full channel, including part of domain III, the entire domain IV, and the cytosolic C-terminus. Loss of *S10s* expression was found to slow cardiac conduction in mice and reduce *I*_Na_ in isolated cardiomyocytes, while overexpression of *S10s* in HEK293 cells stably expressing *SCN5A* increased *I*_Na_.^19^

In the current study, we evaluated the potential of *S10s* as a gene therapy target for treating cardiac conduction abnormalities and associated arrhythmias (*Structured Graphical Abstract*). First, we studied the effect of *S10s* on cellular electrophysiology (EP) and found that *S10s* overexpression increased *I*_Na_ density without changing the gating properties. Consequently, it rescued the phenotypes of *Scn5a*-haploinsufficient cardiomyocytes. Second, we observed that *S10s* gene therapy improved ventricular conduction and rescued conduction slowing in *Scn5a*-haploinsufficient mice. Third, we showed that *S10s* gene therapy reduced susceptibility to cardiac arrhythmias in a reperfusion arrhythmia mouse model. Finally, in an effort to work towards human application, we also demonstrated *S10s*-induced augmentation of *I*_Na_ in human induced pluripotent stem cell-derived cardiomyocytes (hiPSC-CMs), and its anti-arrhythmic effects using *in silico* human cardiac tissue and whole-heart models.^20–22^

## Methods

A detailed description of the methods is provided in the Supplementary material.

## Results

### *S10s* gene therapy rescues peak *I*_Na_ in *Scn5a^+/Δ7bp^* mouse cardiomyocytes

**Figure 1:**
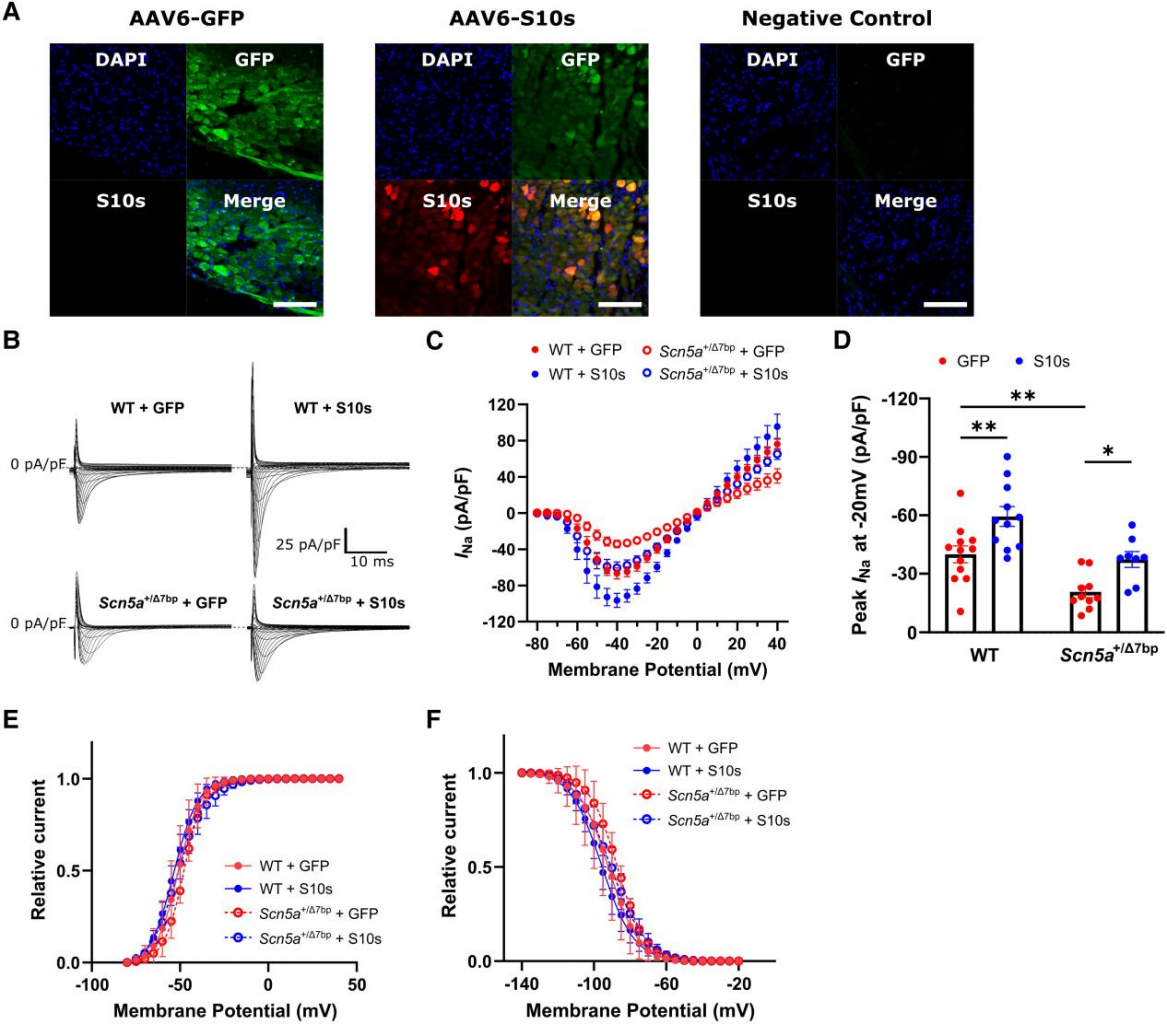
*S10s* gene therapy increases sodium current density in cardiomyocytes isolated from wild type and *Scn5a*^+/Δ7bp^ mice. (*A*) Immunofluorescence staining images of P2A-tagged S10s in mouse left ventricles. GFP expression was observed in mice injected with AAV6-GFP and AAV6-S10s while S10s expression was only observed in mice injected with AAV6-S10s. Remote non-injected site was used as negative control. Scale bars represent 200 µm. (*B*) Typical sodium currents (*I*_Na_) in cardiomyocytes isolated from mice injected with AAV6-GFP or AAV6-S10s. Each group contains 8–12 cells isolated from 3 to 4 mice. (*C*) Average current–voltage relationships of *I*_Na_ density. (*D*) Peak *I*_Na_ at −20 mV. (*E*) Activation curves of *I*_Na_. (*F*) Inactivation curves of *I*_Na_. Data are presented as mean ± SEM. Data were compared using two-way ANOVA with *post hoc* Fisher’s Least Significant Difference (LSD) test. *P < .05; **P < .01

We started our evaluation of *S10s* overexpression with cellular studies. In order to deliver *S10s* to cardiomyocytes, we cloned human *S10s* into a bicistronic expression cassette, which contains a self-cleaving P2A-GFP as a fluorescent marker, allowing identification and isolation of transduced cells for cellular EP studies (see Supplementary data online, *Figure S1*). The control vector expresses GFP alone. AAV serotype 6-S10s-P2A-GFP (AAV6-S10s) and AAV6-GFP vectors were produced (see Supplementary data online, *Figure S1C*, top) and injected in the apex of mouse hearts via intramyocardial injection, at a dose of 1 × 10^11^ viral genome (VG)/mouse. Immunofluorescence staining confirmed the successful overexpression of S10s and GFP in the injected mice (*Figure 1A*). Haematoxylin and eosin (H&E) staining images showed moderate (immune) cell accumulation at the injection sites in both control and S10s groups (see Supplementary data online, *Figure S2*), suggesting a transgene-independent immune response.

We then performed whole-cell patch-clamp studies on GFP-positive ventricular cardiomyocytes isolated from AAV-injected mice 2 weeks post-injection to measure *I*_Na_. Both wild type (WT) mice and mice heterozygous for a 7-bp deletion in *SCN5A* exon 3 causing haploinsufficiency (*Scn5a^+/Δ7bp^*) were used.^23^ Typical *I*_Na_ recordings are shown in *Figure 1B* and the average current–voltage relationships are shown in *Figure 1C*. *Scn5a^+/Δ7bp^* mice showed significantly smaller peak *I*_Na_ density compared to WT mice [at −20 mV: WT −40.1 pA/pF vs. *Scn5a^+/Δ7bp^* −20.8 pA/pF; predicted mean difference (PMD) −19.3; 95% confidence interval (CI) −31.2 to −7.36; *P* = .0022], when both injected with control vectors (*Figure 1C* and *D*), confirming the phenotype of *Scn5a*-haploinsufficiency. In WT mice, *S10s* increased peak *I*_Na_ density at −20 mV by 48.4% (GFP −40.1 pA/pF vs. S10s −59.5 pA/pF; PMD 19.4; 95% CI 7.79 to 31.1; *P* = .0017) (*Figure 1D*). In *Scn5a^+/Δ7bp^* mice, *S10s* increased peak *I*_Na_ density at −20 mV by 80.4% (GFP −20.8 pA/pF vs. S10s −37.5 pA/pF; PMD 16.7; 95% CI 3.49 to 29.9; *P* = .015) (*Figure 1D*), restoring peak *I*_Na_ density to WT levels. In both WT and *Scn5a^+/Δ7bp^* mice, the time constant of the fast and slow inactivating components and the amplitude ratio of the slow inactivation components were not significantly different (see Supplementary data online, *Figure S3A*–*C*). Moreover, overexpression of *S10s* did not affect voltage-dependency of activation or inactivation, as indicated by the overlapping curves that were fitted to the individual (in)activation data (*Figure 1E* and *F*), and by the absence of significant differences in their half-(in)activation voltages and slope factors (see Supplementary data online, *Figure S3D*). These findings indicated that *S10s* overexpression increases peak *I*_Na_ in *Scn5a*^+/Δ7bp^ mouse cardiomyocytes and successfully restores it to WT level.

### *S10s* gene therapy restores action potential properties in *Scn5a*^+/Δ7bp^ mouse cardiomyocytes

**Figure 2:**
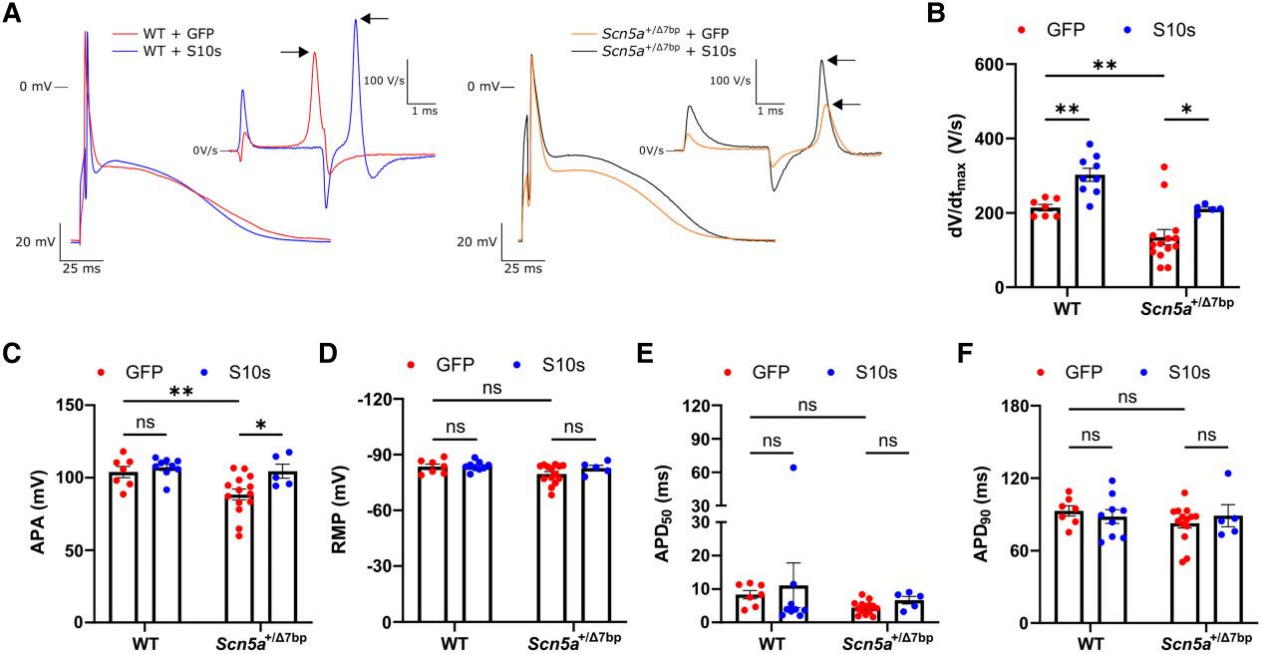
*S10s* gene therapy rescues the phenotypes of *Scn5a*^+/Δ7bp^ cardiomyocytes. (*A*) Typical examples of action potentials (APs) elicited at 6 Hz stimulation and their time derivatives near the upstroke (insets) of cardiomyocytes isolated from mice injected with AAV6-GFP or AAV6-S10s. Arrows indicate the maximal action potential upstroke velocity (d*V*/d*t*_max_). (*B–F*) Action potential parameters of cardiomyocytes isolated from mice injected with AAV6-GFP or AAV6-S10s. Each group contains 5–14 cells isolated from 3 to 4 mice. (*B*) d*V*/d*t*_max_. (*C*) AP amplitude (APA). (*D*) Resting membrane potential (RMP). (*E*) AP duration at 50% of repolarization (APD_50_). (*F*) AP duration at 90% of repolarization (APD_90_). Data are presented as mean ± SEM. Data were compared using two-way ANOVA with *post hoc* Fisher’s LSD test. *P < .05, **P < .01; ns, not significant

To further characterize the cellular EP effect of *S10s* overexpression, we recorded APs (typical examples are shown in *Figure 2A*). Compared to WT cells, *Scn5a^+/Δ7bp^* cells showed significantly slower maximal AP upstroke velocity (d*V*/d*t*_max_; WT 214 V/s vs. *Scn5a^+/Δ7bp^* 135 V/s; PMD 79.4; 95% CI 25.5 to 133; *P* = .0052) and smaller AP amplitude (APA) (WT 104 mV vs. *Scn5a^+/Δ7bp^* 88.4 mV; PMD 15.6; 95% CI 4.88 to 26.3; *P* = .0057), when injected with control vectors (*Figure 2B* and *C*). No significant differences were observed in resting membrane potential (RMP; *Figure 2D*) or APDs (*Figure 2E* and *F*). In WT cells, *S10s* overexpression significantly increased d*V*/d*t*_max_ (*Figure 2B*). In *Scn5a^+/Δ7bp^* cells, *S10s* overexpression rescued the phenotypes by increasing both d*V*/d*t*_max_ (GFP 135 V/s vs. S10s 211 V/s; PMD −76.1; 95% CI −137 to −15.3; *P* = .016) and APA (GFP 88.4 mV vs. S10s 104 mV; PMD −16.1; 95% CI −28.2 to −4.08; *P* = .010). *S10s* overexpression did not change the RMP or APDs in either WT or *Scn5a^+/Δ7bp^* cells (*Figure 2D–F*), indicating that ionic currents other than *I*_Na_ were virtually unaffected. To study the potential side-effect of AAV transduction on cellular EP, we also recorded APs from cells of untreated WT mice and found no significant differences in all measured parameters, when compared to those injected with control vectors (see Supplementary data online, *Figure S4*). These data demonstrated that *Scn5a^+/Δ7bp^* cardiomyocytes exhibited phenotypes of slower d*V*/d*t*_max_ and smaller APA, which *S10s* gene therapy successfully rescued by increasing *I*_Na_ without affecting AP parameters other than d*V*/d*t*_max_ and APA.

### *S10s* gene therapy increases cardiac conduction velocity in WT mice

Given the importance of *I*_Na_ to cardiac impulse propagation,^24^ we hypothesized that the *S10s-*induced increase in *I*_Na_ density would enhance cardiac conduction velocity (CV) at the whole organ level.

**Figure 3:**
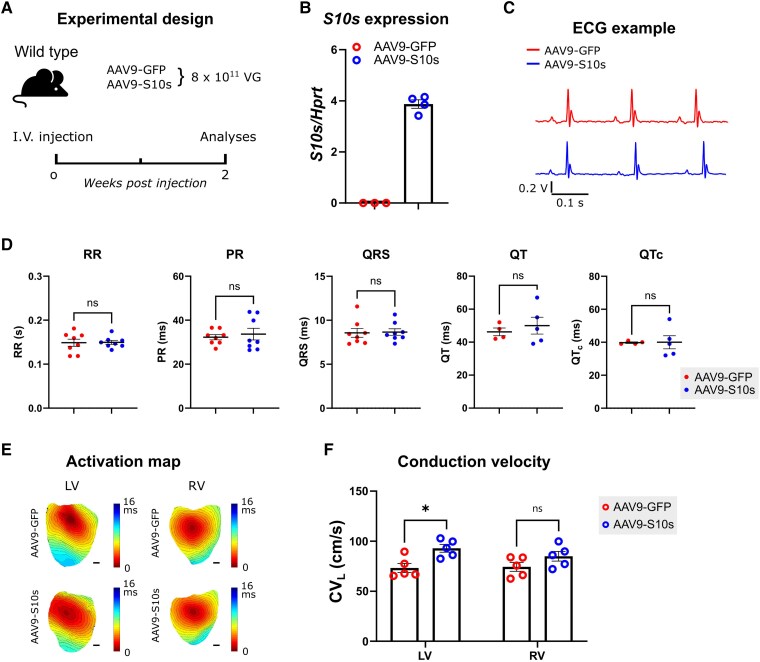
*S10s* gene therapy increases cardiac conduction velocity in wild type mice. (*A*) Schematic diagram of the experimental design. (*B*) mRNA expression level of *S10s* in left ventricles of wild type mice injected with AAV9-GFP or AAV9-S10s. (*C*) Typical ECG traces. (*D*) Average ECG parameters. (*E*) Representative activation maps of ventricles. Scale bars represent 1 mm. (*F*) Epicardial longitudinal conduction velocity (CV_L_) measured from ventricles stimulated at 8 Hz. Data are presented as mean ± SEM. Data were compared using Student’s *t*-test (*D*) or two-way ANOVA with *post hoc* Fisher’s LSD test (*F*). *P < .05; ns, not significant

In order to achieve homogenous *S10s* overexpression throughout the heart, we adapted our existing AAV delivery vector in two ways: (i) the ubiquitous CMV promoter was replaced by a cardiac-specific troponin T (cTnT) promoter and (ii) we made use of the AAV cardio-tropic serotype 9 (AAV9), instead of AAV6 (see Supplementary data online, *Figure S1C*, bottom). We first administered the AAV vectors to 6-week-old WT mice via intravenous injection at a dose of 8 × 10^11^ VG/mouse (*Figure 3A*). Overexpression of *S10s* in left ventricular tissue of AAV9-S10s mice was detected by RT-qPCR (*Figure 3B*). Two weeks post-injection, surface ECG analyses showed no significant differences in RR, PR, QRS, QT, and QTc intervals between AAV9-S10s mice and AAV9-GFP mice (*Figure 3C* and *D*). Optical mapping on isolated hearts revealed significantly increased longitudinal CV (CV_L_) in the left ventricle of AAV9-S10s mice, when compared to AAV9-GFP mice (*Figure 3E* and *F*). Transversal CV (CV_T_) in the left ventricle was not affected by *S10s* (see Supplementary data online, *Figure S5A*), and consequently the anisotropic ratio was significantly increased by *S10s* (see Supplementary data online, *Figure S5D*). *S10s* did not affect CV_L_ or CV_T_ in the right ventricle (*Figure 3F* and Supplementary data online, *Figure S5A*). H&E staining did not reveal structural differences between hearts of control and S10s treated mice (see Supplementary data online, *Figure S2B*). These data support the concept that *S10s* overexpression increases ventricular CV_L_ in WT mice by augmenting *I*_Na_.

### *S10s* gene therapy rescues conduction slowing in *Scn5a^+/Δ7bp^* mice

**Figure 4:**
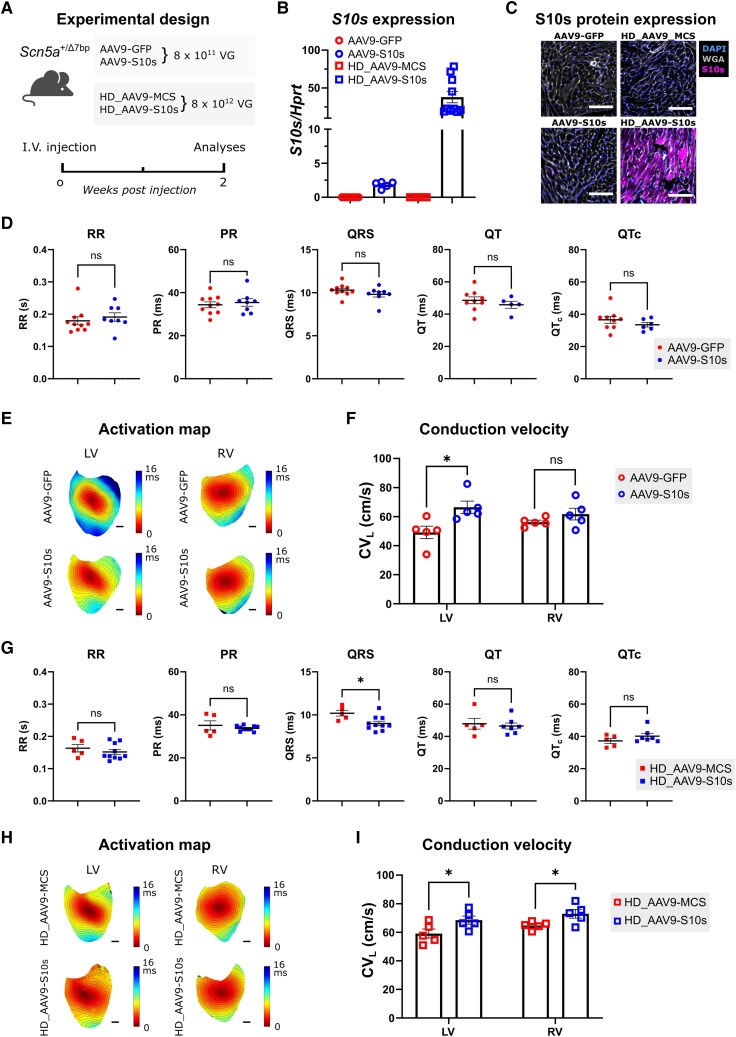
*S10s* gene therapy improves cardiac conduction in *Scn5a^+/Δ7bp^* mice. (*A*) Schematic diagram of the experimental design. (*B*) mRNA expression level of *S10s* in left ventricles of *Scn5a^+/Δ7bp^* mice injected with AAV vectors. (*C*) Immunofluorescence staining images of P2A-tagged S10s. S10s expression was detected in HD_AAV9-S10s injected hearts. Scale bars represent 200 µm. (*D*) Average ECG parameters from *Scn5a^+/Δ7bp^* mice injected with AAV9-GFP or AAV9-S10s. (*E*) Representative activation maps of ventricles. Scale bars represent 1 mm. (*F*) Epicardial longitudinal conduction velocity (CV_L_) measured from ventricles stimulated at 8 Hz. (*G*) Average ECG parameters from *Scn5a^+/Δ7bp^* mice injected with HD_AAV9-MCS or HD_AAV9-S10s. (*H*) Representative activation maps of ventricles. Scale bars represent 1 mm. (*I*) Epicardial CV_L_ measured from ventricles stimulated at 8 Hz. Data are presented as mean ± SEM. Data were compared using Student’s *t*-test (*D* and *G*) or two-way ANOVA with *post hoc* Fisher’s LSD test (*F* and *I*). *P < .05; ns, not significant

We next tested if *S10s* overexpression could rescue the conduction slowing observed in *Scn5a^+/Δ7bp^* mice. AAV9-GFP or AAV9-S10s were administered to *Scn5a^+/Δ7bp^* mice via intravenous injection at an intermediate dose of 8 × 10^11^ VG/mouse (*Figure 4A*). Overexpression of *S10s* in left ventricular tissue of AAV9-S10s mice was detected by RT-qPCR (*Figure 4B*). S10s protein was not detected by immunofluorescence staining (*Figure 4C*), probably because the expression level was below the detection threshold of the anti-P2A antibody. In order to increase the expression level, we injected *Scn5a^+/Δ7bp^* mice with AAV9-S10s at a high dose of 8 × 10^12^ VG/mouse (*Figure 4A*). *S10s* expression was detected in heart and liver by RT-qPCR (*Figure 4B* and Supplementary data online, *Figure S6A*). Cardiac *S10s* expression was approximately 20-fold higher in mice of the high dose group compared to that in mice of the intermediate dose group (*Figure 4B*). Immunofluorescence staining showed clear S10s protein expression in the left ventricles (*Figure 4C*). Mice injected with empty vectors (AAV9-MCS) at the same dose were used as control (*Figure 4A* and Supplementary data online, *Figure S1C*). This was to avoid the potential adverse effect of high GFP expression on cardiac conduction since GFP has previously been shown to negatively affect CV in neonatal rat cardiomyocytes cultures.^25^ H&E staining did not reveal structural differences between hearts of control, intermediate and high dose-treated S10s mice (see Supplementary data online, *Figure S2C* and *D*).

Two weeks post-injection, electrocardiograms (ECGs) were recorded *in vivo* and hearts were isolated for optical mapping. In the intermediate dose groups, surface ECG analyses showed no significant changes in RR, PR, QRS, QT, and QTc intervals (*Figure 4D*). Optical mapping revealed that AAV9-S10s gene therapy significantly increased CV_L_ in the left ventricle compared to control (*Figure 4E* and *F*). CV_T_ and CV_L_/CV_T_ ratio were not changed by *S10s* (see Supplementary data online, *Figure S5B* and *E*). In the high dose groups, surface ECG analyses revealed significantly shortened QRS interval in high dose AAV9-S10s mice comparing to control (MCS 10.2 ms vs. S10s 9.01 ms; PMD −1.19; 95% CI −2.15 to −0.235; *P* = .018) (*Figure 4G*). No significant changes were observed between the high dose groups in regards of RR, PR, QT, and QTc intervals (*Figure 4G*). Optical mapping showed that AAV9-S10s gene therapy at high dose significantly increased CV_L_ in both left (MCS 59.1 cm/s vs. S10s 68.5 cm/s; PMD −9.37; 95% CI −17.4 to −1.31; *P* = .025) and right ventricles (MCS 64.7 cm/s vs. S10s 72.9 cm/s; PMD −8.21; 95% CI −16.3 to −0.154; *P* = .046) (*Figure 4H* and *I*), when compared to control. CV_T_ and CV_L_/CV_T_ ratio were not significantly different (see Supplementary data online, *Figure S5C* and *F*). Moreover, AAV9-S10s gene therapy at high dose rescued the conduction slowing phenotype of *Scn5a^+/Δ7bp^* mice. *Scn5a^+/Δ7bp^* mice injected with high dose AAV9-S10s were at similar levels as WT mice injected with AAV9-GFP in QRS interval and ventricular CV_L_. These data showed that *S10s* gene therapy improved cardiac conduction and rescued the conduction slowing phenotype in *Scn5a^+/Δ7bp^* mice.

### *S10s* gene therapy protects against VT in mouse hearts

**Figure 5:**
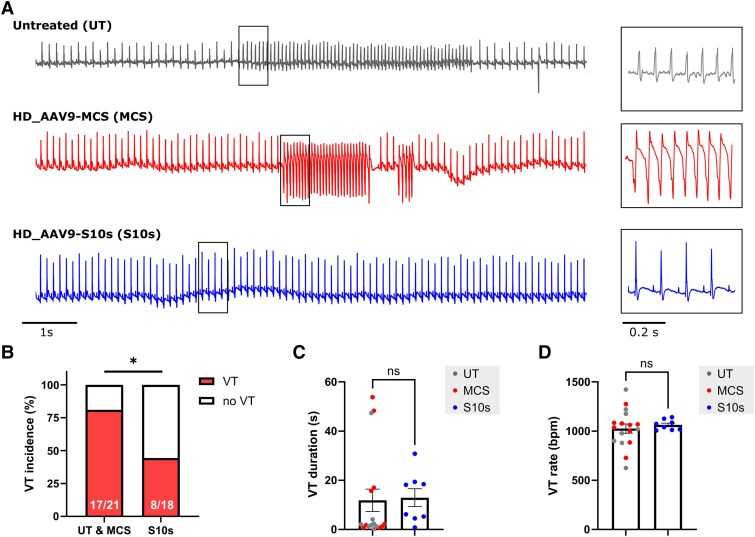
*S10s* gene therapy protects against ventricular tachycardia in mice. (*A*) Typical ECG tracing of ventricular tachycardia induction following ischaemia–reperfusion injury. Higher magnification tracings are provided on the right. (*B*) Incidence of ventricular tachycardia. Number of animals with ventricular tachycardia and total number of animals studied are presented within the associated bar charts. (*C*) Total ventricular tachycardia duration per animal. (*D*) Average ventricular tachycardia rate per animal. Data are presented as percentage (*B*) or mean ± SEM (*C* and *D*). Data were compared using Fisher’s exact test (*B*) or Student’s *t*-test (*C* and *D*). Untreated and HD_AAV9-MCS groups were first compared and later combined into one control group, as no significant differences were found between untreated and HD_AAV9-MCS groups.*P < .05; ns, not significant

Conduction abnormalities predispose the heart to unidirectional block, which is a prerequisite for re-entrant arrhythmias, the predominant mechanism of malignant ventricular arrhythmias.^26,27^ We therefore hypothesized that *S10s* gene therapy can be employed to normalize conduction and thereby reduce susceptibility to re-entrant arrhythmias. We tested our hypothesis in an ischaemia–reperfusion (I/R) arrhythmia mouse model, in which re-entry activity was suggested as a key factor for arrhythmia genesis and sodium channel overexpression has been shown to suppress VT.^28^ Before the controlled application of I/R injury, mice were either untreated, injected with AAV9-MCS, or injected with AAV9-S10s at high dose. I/R-induced arrhythmia testing was subsequently performed 4 weeks later. *Figure 5A* illustrates representative tracings from each group. Untreated and AAV9-MCS injected mice had a VT incidence of 80.0% (*n* = 10) and 81.8% (*n* = 11), respectively. Because no significant differences were expected and detected between these two groups (UT 80.0% vs. MCS 81.8%; odds ratio 0.889; 95% CI 0.119 to 6.74, *P* > .99), we bundled them into one control group (81.0%, *n* = 21) and used this for our comparison with the AAV9-S10s group. In AAV9-S10s injected mice, VT inducibility was reduced to 44.4% (*n* = 18), significantly lower than that in control mice (control 81.0% vs. S10s 44.4%; odds ratio 5.31; 95% CI 1.27 to 18.3; *P* = .024) (*Figure 5B*). No differences were observed in the duration of VT or the rate of VT (*Figure 5C* and *D*).

We also examined heart weight, blood chemistry and gene expression profile from mice injected with AAV9-S10s at high dose to study the safety profile of *S10s* gene therapy at 4 weeks of follow-up. Heart weight, blood AST, ALT, CK-MB, and CRP levels were not changed by *S10s* gene therapy (see Supplementary data online, *Table S1*). Blood ALP levels of AAV9-S10s injected mice were significantly lower than those of untreated mice, but not different from AAV9-MCS injected mice (see Supplementary data online, *Table S1*). Gene expression of selected ion channels and connexins also remained unchanged 4 weeks post-injection (see Supplementary data online, *Figure S7*). These data demonstrated the anti-arrhythmic effects and long-term safety of *S10s* gene therapy in mice.

### *S10s* overexpression increases d*V*/d*t*_max_ in hiPSC-CMs

**Figure 6:**
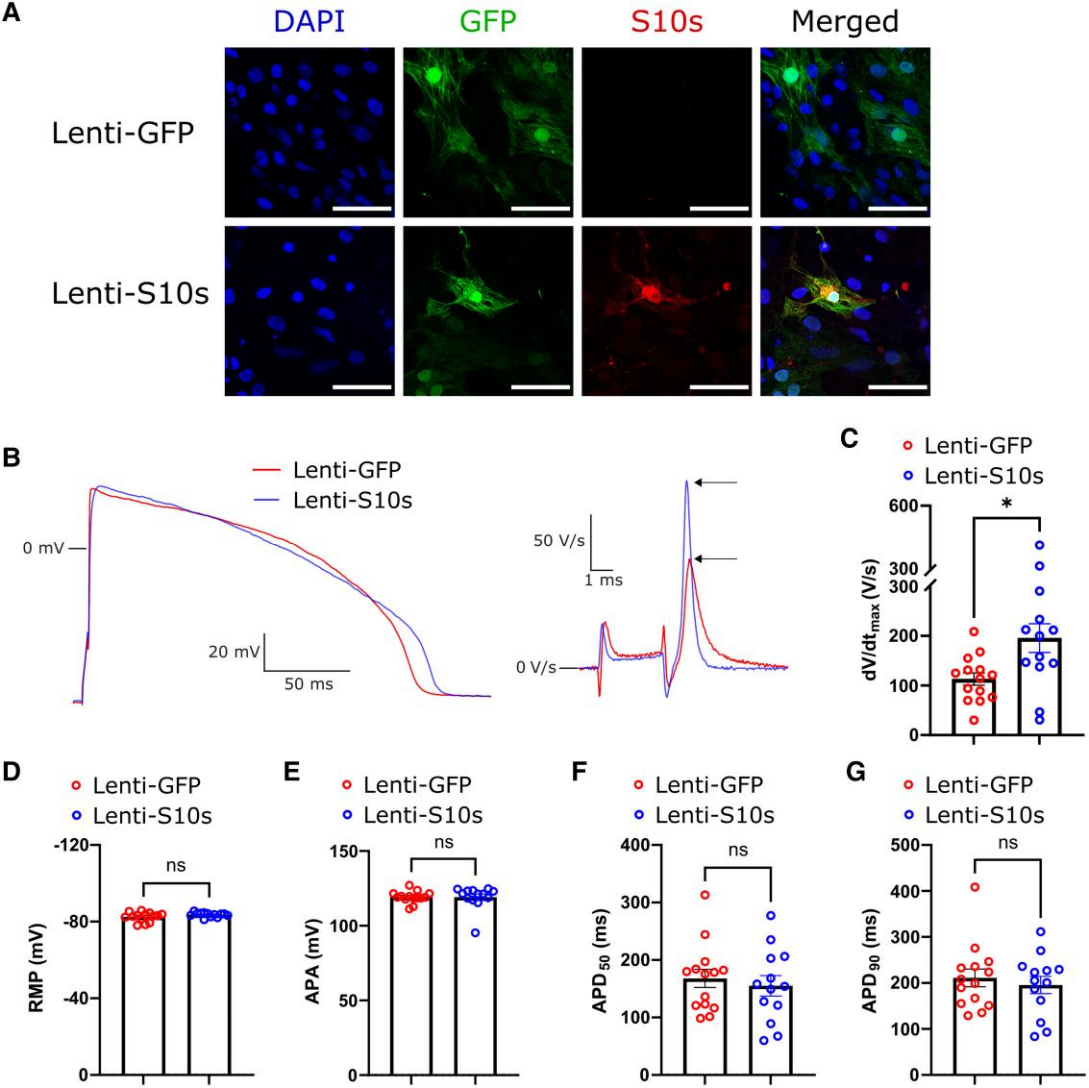
*S10s* overexpression increases action potential upstroke velocity in human iPSC-derived cardiomyocytes. (*A*) Immunofluorescence staining images of GFP and P2A-tagged S10s in hiPSC-CMs. S10s expression was detected in hiPSC-CMs transduced with Lenti-S10s but not in hiPSC-CMs transduced with Lenti-GFP. Scale bars represent 50 µm. (*B*) Typical examples of action potentials elicited at 1 Hz (left) and their time derivatives near the upstroke (right) from hiPSC-CMs transduced with Lenti-GFP or Lenti-S10s. Arrows indicate the d*V*/d*t*_max_. (*C–G*) d*V*/d*t*_max_, RMP, APA, APD_50_) and APD_90_ from hiPSC-CMs transduced with Lenti-GFP or Lenti-S10s. Each group contains 13–14 cells from three differentiations. Data are presented as mean ± SEM. Data were compared using Student’s *t*-test. *P < .05; ns, not significant

In order to study the impact of S10s on EP properties of human cardiomyocytes, we made use of hiPSC-CMs. For optimal transduction efficiency, we transduced hiPSC-CMs with lentiviral vectors containing either the S10s-GFP bicistronic expression cassette (Lenti-S10s) or GFP alone (Lenti-GFP) (see Supplementary data online, *Figure S1C*, top). Successful delivery of *S10s* was confirmed by immunofluorescence staining on cells (*Figure 6A*). Transduced hiPSC-CMs were used for AP measurements with the patch-clamp methodology, 5–7 days post-transduction (*Figure 6B*). Dynamic clamp was used to inject a 2 pA/pF Kir2.1-like current to overcome the depolarized and spontaneous state of the hiPSC-CMs which hampers the functional availability of Na_V_1.5 channels (for reviews see ref. 29,30, and primary references cited therein).^29–32^ Similar to our results in mouse cardiomyocytes (*Figure 2*), *S10s* overexpression led to significantly increased d*V*/d*t*_max_ with a 73% increase compared to the GFP group (GFP 113 V/s vs. S10s 196 V/s; PMD 82.5; 95% CI 19.1 to 146; *P* = .013) (*Figure 6C*). No significant differences were observed in RMP, APA, and APDs between the groups (*Figure 6D–G*).

### *S10s* overexpression increases excitability and CV in simulated human tissues

Based on our results that *S10s* overexpression increases CV in *Scn5a^+/Δ7bp^* mice, and that it increases d*V*/d*t*_max_ in hiPSC-CMs, we hypothesized that introduction of *S10s* into human ventricular tissues could increase CV in *SCN5A*-haploinsufficient background and prevent conduction block.

**Figure 7:**
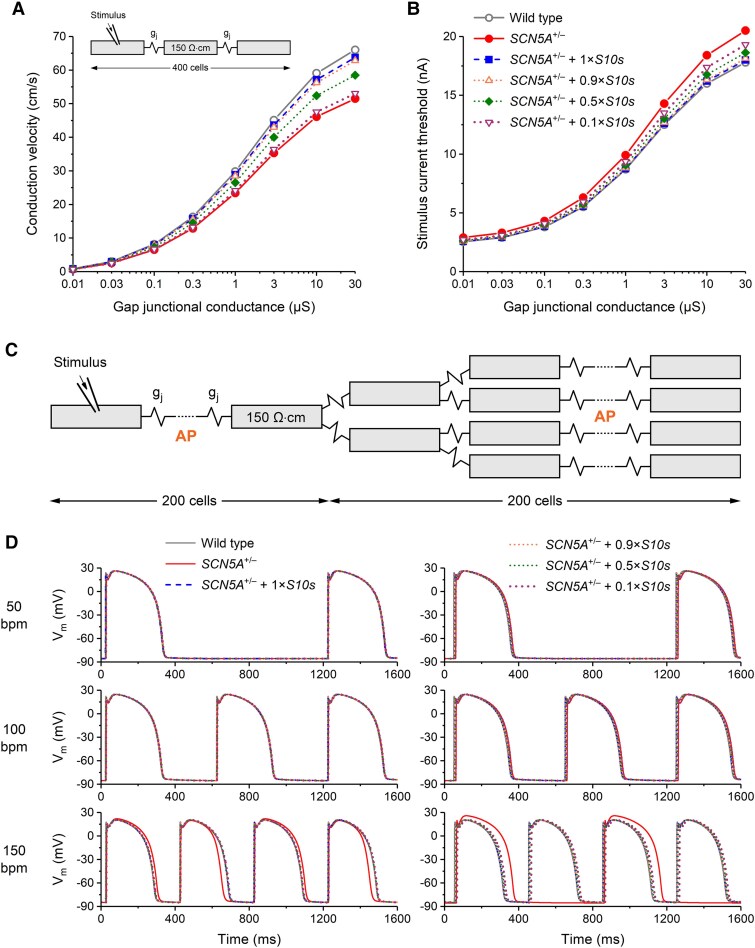
*S10s* gene therapy improves conduction and increases excitability in a simulated strand of human left ventricular cardiomyocytes. (*A*) Longitudinal conduction velocity in a simulated linear strand of human left ventricular cardiomyocytes as a function of gap junctional conductance (*g_j_*). Complete loss-of-function mutation in *SCN5A* simulated by a 50% decrease in Na_V_1.5 conductance (‘*SCN5A*^+/−^’) and the effect of *S10s*, provided 100% successful transduction, as an 80.4% increase in remaining Na_v_1.5 conductance to 90.2% of the wild type Na_v_1.5 conductance (‘*SCN5A*^+/−^ + 1*×S10s*’). Data for 0.9×, 0.5×, and 0.1× *S10s* overexpression levels obtained by scaling the 80.4% increase accordingly. Individual cells of the 400-cell strand (inset) described according to the human left ventricular cell model by Ten Tusscher *et al*.,^20^ as updated by Ten Tusscher and Panfilov.^21^ Myoplasmic resistivity set to 150 Ω·cm.^33^ (*B*) Stimulus current threshold of the 400-cell strand as a function of g_j_. (*C*) Structure of the 400-cell strand with branches at cells #200 and #201 of the strand used to simulate an increased electrical load. *g_j_* set to 10 µS in the associated simulations. (*D*) Action potential conduction in the branched strand of (C) at stimulus rates of 50 (top), 100 (middle), and 150 beats/min (bottom). Action potentials of cells #100 (left) and #300 (right) of the strand, as indicated by ‘AP’ in (C), under control conditions (wild type) and in case of *SCN5A*^+/−^ and *SCN5A*^+/−^ + 1×*S10s*. Action potentials in case of 0.9×, 0.5×, and 0.1× *S10s* overexpression levels are also shown

We performed *in silico* experiments using computer-simulated 1D strands of human left ventricular cardiomyocytes to test our hypothesis. Four hundred cells with a myoplasmic resistivity of 150 Ω·cm were electrically coupled through a gap junctional conductance g_j_ and stimulated at one end of the strand with a square stimulus of 2 ms duration at a rate of 1 Hz (*Figure 7A*, inset).^20,21^ In this *in silico* tissue model, a heterozygous loss-of-function mutation in *SCN5A* was simulated by a 50% decrease in Na_V_1.5 conductance (labelled *SCN5A*^+/−^), representing an entirely non-functional *SCN5A* mutation. We modelled the effect of *S10s* at ‘normal’ (1×) overexpression level with an 80.4% increase in the residual Na_V_1.5 conductance to 90.2% of the WT Na_V_1.5 conductance in each of the cardiomyocytes (labelled *SCN5A*^+/−^ + 1*×S10s*). This 80.4% increase was chosen according to the average increase in peak *I*_Na_ density observed in our voltage clamp experiments of isolated murine cardiomyocytes in *Figure 1*.

As illustrated in *Figure 7A* (solid and dashed lines), modelling heterozygosity of the complete loss-of-function mutation in *SCN5A* resulted in a 17–22% decrease in CV over a wide range of *g_j_* values, which was reduced to 3–4% upon the simulated application of *S10s* (with a transduction efficiency of 100%). This decrease in CV amounted to 4%–5%, 9%–11%, and 15%–20% when the *S10s*-induced increase in peak *I*_Na_ density was lowered by 10%, 50%, and 90%, respectively (mimicking 0.9×, 0.5×, and 0.1× *S10s* overexpression levels) (*Figure 7A*, dotted lines). At the same time, the mutation-induced decrease in excitability, expressed as increase in stimulus current threshold, amounted to ≈15% in absence of *S10s*, which was reduced to only ≈1% in presence of *S10s* at 1× overexpression level (*Figure 7B*, solid and dashed lines), further indicating that excitability could be largely restored by *S10s*. This increase in stimulus current threshold amounted to ≈2%, 4%–5%, and 7%–8% in presence of *S10s* at 0.9×, 0.5×, and 0.1× overexpression levels, respectively (*Figure 7B*, dotted lines).

We also constructed a linear strand with multiple branches and g_j_ set to 10 µS^33^ (*Figure 7C*) that was stimulated at rates of 50–150 b.p.m. In this more demanding setting, the mutant *I*_Na_ elicited regular APs in the unbranched part of the strand at 50 and 100 b.p.m. (*Figure 7D*, left, top and middle panels) that were successfully conducted to the branched part of the strand (*Figure 7D*, right, top and middle panels). However, the mutant *I*_Na_ appeared unable to do so at 150 b.p.m. (*Figure 7D*, bottom panels). At this higher rate, every other AP in the unbranched part of the strand was not completely full-blown, resulting in alternating long-short APs (*Figure 7D*, left, bottom panel). The short AP was not successfully conducted to the branched part of the strand and this partial block thus resulted in a 2:1 conduction pattern in the strand (*Figure 7D*, right, bottom panel). Of note, such irregularities were not observed in the unbranched strand (data not shown) and in the case of antidromic propagation (see Supplementary data online, *Figure S8*). Application of *S10s* completely restored successful conduction, not only at 1× *S10s* overexpression level (*Figure 7D*, dashed lines) but also at significantly lower *S10s* overexpression levels (*Figure 7D*, dotted lines).

**Figure 8:**
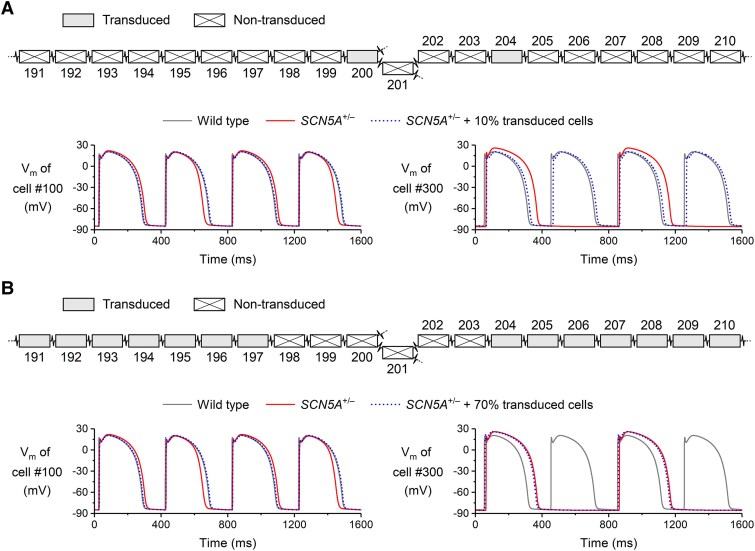
Evaluation of *S10s* gene therapy in a setting of non-uniform transduction. (*A,B*) Configuration of transduced vs. non-transduced cells in the strand (top) and associated action potential conduction at 150 b.p.m. (bottom). (*A*) Successful rescue of the 2:1 block upon transduction of the cells at positions 200 and 204. (*B*) Rescue fails when none of the cells near the splits are transduced. Transduction simulated by increasing the Na_V_1.5 conductance of the transduced cell to 90.2% of its WT level (i.e. the average level observed in the voltage clamp experiments of *Figure 1*)

Although even at 0.1× overexpression level *S10s* appeared sufficient to rescue the 2:1 block that was observed at 150 b.p.m. (*Figure 7D*), it should be recognized that this result was obtained at a simulated transduction efficiency of 100%. In a more realistic setting, with a non-uniform mix of transduced and non-transduced cells, the result depends on the status of the cells (transduced vs. non-transduced) close to the splits in the strand, as shown in *Figure 8*. In the configuration of *Figure 8A* (top), only two nearby cells, at positions 200 and 204, are transduced (each showing the average increase in their Na_V_1.5 conductance to 90.2% of the WT level, as observed in our voltage clamp experiments of *Figure 1*). Yet, the 2:1 block at 150 b.p.m. is still successfully rescued (*Figure 8A*, bottom). Conversely, in the configuration of *Figure 8B* (top), where many more cells are transduced throughout the strand but not near the splits, the rescue fails (*Figure 8B*, bottom). For the simulations of *Figure 8A* and *B*, the status of the cells 1–190 and 211–400 (transduced vs. non-transduced) was assigned in a similar pattern (compared to that shown in the configurations of *Figure 8A* and *B*), so that the overall transduction efficiency was 10% in panel A and 70% in panel B. However, the exact pattern in the remaining parts of the strand does not seriously affect the results of the simulations (data not shown).

Altogether, these results illustrate that *S10s* can prevent unidirectional conduction block at high stimulation frequencies. Such effect may be achieved already with a modest transduction efficiency, provided that the critical cells are effectively transduced. Therefore, it appears desirable to aim for a high local transduction efficiency to assure a high probability for correction of unidirectional block (as shown in *Figure 7*).

### *S10s* overexpression reduces VT inducibility in a computational whole-heart model with *SCN5A* loss-of-function mutations

Following our demonstration of *S10s*’s capability to restore Na_V_1.5 and increase CV and to prevent conduction block in computer-simulated 1D strands of human ventricular tissue, we advanced our investigation to a more complex and clinically relevant scenario. We hypothesize that *S10s* gene therapy has the potential to mitigate arrhythmia induction in a human heart affected by Na_V_1.5 dysfunction. To test this hypothesis, we employed a 3D image-based computational whole-heart model and compared VT inducibility before and after the digital application of *S10s* gene therapy.

**Figure 9:**
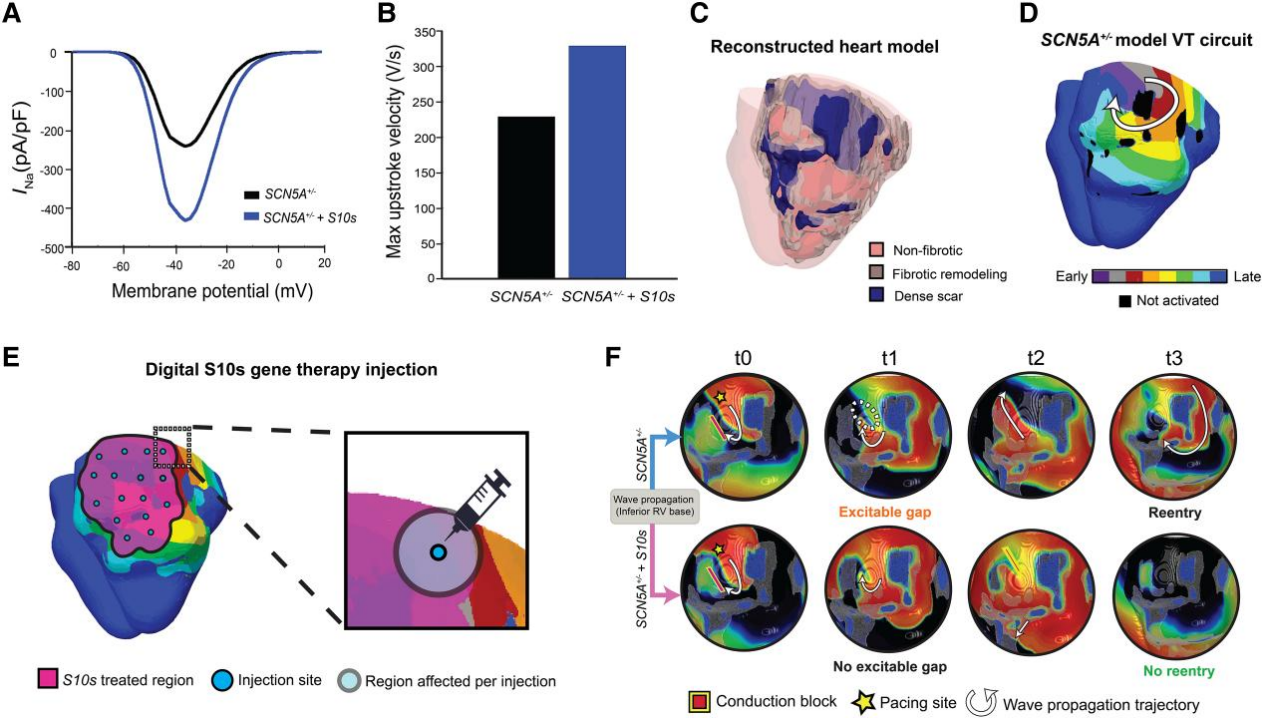
*S10s* suppresses arrhythmia induction in a 3D human whole-heart model. (*A*) Current–voltage relationships of *I*_Na_. (*B*) The d*V*/d*t*_max_ of the steady-state action potentials at 1 Hz pacing. (*C*) Reconstructed ventricular model with three different tissue types: non-fibrotic myocardium (pink), fibrotic remodelling (grey) and dense scar (dark blue). Scar region is non-conductive; the non-fibrotic myocardium exhibits altered electrophysiological (EP) properties due to the *SCN5A* mutation, while the fibrotic regions include additional fibrosis-induced changes in electrophysiological properties. (*D*) Activation patterns of the ventricular tachycardia re-entrant circuit induced by validated *in silico* rapid pacing protocol at the base of the *SCN5A^+/−^* heart model. (*E*) Schematics describing digital *S10s* treatment delivery method. The magenta patch indicates the region affected by gene therapy, with the spatial distribution of simulated injection sites visualized in cyan colour. In the right close-up figure, the semi-transparent circle represents a 1-cm radius region affected by each simulated injection. In total, 16 injections were applied to ensure full transmural coverage of the region associated with the ventricular tachycardia circuit. (*F*) Comparison of wavefront propagation following rapid pacing in the heart model before (top) and after (bottom) *S10s* gene therapy is applied. Each row shows a series of frames that depict the continuous wave propagation in a portion of the 3D whole-heart model. The images in each column show the same instant of the simulation. Yellow stars stand for the pacing location; red lines with yellow contours mark the conduction block. Electrical wave propagation trajectories were marked in white arrows. The dotted region marks the location of excitable gap that occurred at t1. For the *SCN5A^+/−^* model, a sustained re-entry was induced. For the *SCN5A^+/−^ + S10s* model, no re-entry was induced

Firstly, we developed a novel computational cell model that incorporated the EP properties of *SCN5A* loss-of-function mutation (*SCN5A^+/−^*). To model the effects of *S10s* gene therapy, we augmented the remaining *I*_Na_ by 80.4% in this *SCN5A^+/−^* cell model as previously described, resulting in a post-treatment cell model referred to as *SCN5A*^+/−^ + *S10s*. Compared to the *SCN5A*^+/−^ model, the post-treatment *SCN5A*^+/−^ + *S10s* model has a higher peak current density (*SCN5A*^+/−^ + *S10s* 432 vs. *SCN5A*^+/−^ 242 pA/pF) and a faster upstroke velocity (*SCN5A*^+/−^ + *S10s* 329 vs. *SCN5A*^+/−^ 229 V/s), as shown in *Figure 9A* and *B*.

We then reconstructed the whole-heart model with personalized diffuse fibrosis and dense scar distributions derived from late-gadolinium enhanced magnetic resonance imaging scan, as described in previous publications^22,34^ and depicted in *Figure 9C*. Simulations were conducted to evaluate VT inducibility in the *SCN5A^+/−^* heart using a validated *in silico* rapid pacing protocol, mimicking settings in the clinical electrophysiological studies.^35,36^ From the simulations, a re-entrant circuit was induced in the basal posterolateral wall of RV, as shown in *Figure 9D*. We initially applied *S10s* gene therapy (with a transduction efficiency of 100%) globally to the entire heart and found that this treatment successfully terminated the previously induced VT (data not shown). Next, we investigated whether locally applied *S10s* gene therapy could achieve a similar anti-arrhythmic effect. *S10s* gene therapy was applied with 16 injections to fully cover the region associated with the arrhythmia circuit (*Figure 9E*). The simulation was repeated, and results revealed that after locally applying the *S10s* gene therapy, the previously induced VT got effectively terminated.

To gain a deeper insight into the role of *S10s* in preventing VT induction, we conducted a detailed analysis of the re-entry simulated under *SCN5A^+/−^ and SCN5A^+/−^ + S10s* conditions, as depicted in *Figure 9F*. For both simulations at t0, the wavefronts elicited at the basal-inferior pacing sites encounter preceding wavetails that have been slowed by a region of diffuse fibrosis. This transient conduction block causes the wavefronts to curl clockwise. In the *SCN5A^+/−^* case (*Figure 9F*, top row), the slower CV due to the *I*_Na_ alteration allows the formation of an excitable gap that provides enough time for the tissue to recover and become stimulated again by the curling wave. As a result, a re-entrant circuit evolves and anchors around an adjacent region of dense scar. In contrast, the *SCN5A^+/−^ + S10* case (*Figure 9F*, bottom row) has a recovered *I*_Na_ and CV, allowing the wavefront to curl fast enough to encounter still refractory tissue. This functional block of the curling wavefront prevents the formation of a re-entrant circuit.

## Discussion

Our present work evaluated the potential of *S10s* overexpression as a gene therapy for cardiac arrhythmias that stem from reduction in *I*_Na_. Our data from *ex vivo*, *in vivo*, *in vitro*, and *in silico* experiments provide support for the concept that introduction of *S10s* in ventricular cardiomyocytes increases *I*_Na_, thereby improving cardiac conduction and importantly compensating for *I*_Na_ insufficiency and mitigate the associated risk for arrhythmias.

Reductions in cardiac *I*_Na_ are implicated in various arrhythmia syndromes such as BrS, PCCD, SSS, AF, and scar or cardiomyopathy-related VT/VF.^2–5^ Sodium channel gene transfer could provide an effective therapy to improve cardiac conduction,^37^ yet the development of such an intervention is difficult because the coding sequences of sodium channels exceed the packaging capacity of AAV. Although dual vector systems have been developed to expand AAV packaging capacity,^38^ and *SCN5A* has been successfully implemented in such a system,^9^ translation remains complicated due to the lower intrinsic efficiency (in comparison to a single vector), much higher costs and potential additional safety issues due to the need of a higher dose. Notably, Doisne *et al*. used a high AAV-vector dose in neonatal mice (comparable to our high dose in adult mice),^9^ while cardiac transduction is more easily realized in neonatal mice as compared to adult mice.^11,39,40^ It is therefore reasonable to anticipate that a higher dose is required to achieve a similar effect in adult recipients. As a result, we and others have continued to search for smaller transgenes that can correct sodium channel dysfunction.

The *S10s* transcript was identified in the hearts of mouse and human as a result of investigations into genetic variants associated with conduction abnormalities and BrS, and was shown to represent an endogenous mechanism to modulate Na_V_1.5-mediated *I*_Na_*in vitro* and *in vivo*.^19^ Compared to previous approaches, *S10s* has several advantages as a gene therapy target. First, it has a coding sequence of 2 kb, which easily fits into a single AAV vector, avoiding the use of a dual AAV-vector system. Second, it increases *I*_Na_ density without altering the gating kinetics or APD in cardiomyocytes. This assures APD homogeneity even when it is expressed at varying levels caused by inhomogeneous transduction. Third, *S10s* is only effective in the presence of Na_V_1.5 and does not generate any *I*_Na_ when expressed alone.^19^ This lowers its potential side-effect in case off-target expression occurs. Indeed, we observed hepatic expression of *S10s* transcripts when applied at high dose but found no elevated liver damage parameters in blood chemistry. Finally, as an endogenously expressed gene product in mammals including humans, *S10s* is expected to be well tolerated by its recipients. Nevertheless, the mechanism of action of *S10s* is not fully understood. Because *S10s* does not affect *Scn5a* expression, and the *S10s* gene product co-localizes with Na_V_1.5 on the cell membrane,^19^ it is likely that it increases *I*_Na_ density by physically interacting with Na_V_1.5 proteins. Preliminary *in vitro* data from our lab showed no increase of Na_V_1.5 membrane expression by *S10s* overexpression, suggesting a mechanism other than facilitated trafficking, although further studies are required to gain additional mechanistic insight into the function of *S10s*.

In this study, we found that *S10s* gene therapy significantly increased CV_L_ but not CV_T_ in mouse heart. This might be due to that Na_V_1.5 locates more abundantly in the intercalated disc than in the lateral membrane,^41–43^ since *S10s* is only effective in the presence of Na_V_1.5.^19^ In fact, this different response to sodium channel abundancy between CV_L_ and CV_T_ is not new. Overexpression of Na_V_1.4 in canine heart has been reported to only increase CV_L_ due to its presence in intercalated disks.^44^ Also in two different *Scn5a* heterozygous mouse models, only CV_L_ was significantly decreased but not CV_T_.^23,45^ Furthermore, this increase in CV_L_ in mouse heart was anti-arrhythmic as demonstrated in *Figure 5*.

Loss-of-function mutations in *SCN5A* lead to reduction of *I*_Na_ in cardiomyocytes. This group of mutations can be divided into several categories based on their mechanism of effect. The first category is represented by mutations that generate a premature stop codon by nonsense mutations or frameshifts (e.g. L1393X, C280S*fs61).^46,47^ Such mutations lead to loss of protein expression and often cause more severe phenotypes than others.^48,49^ Our *Scn5a^+/Δ7bp^* mouse model and the simulated human tissue models both represent this category, where the conduction slowing phenotype was largely rescued, demonstrating the usefulness of *S10s* gene therapy for these conditions. The second category is represented by mutations that generate non-functional channels due to gating or permeation defects (e.g. R878C, G1408R)^50,51^ and the third category is represented by mutations that impair the cell surface localization of Na_V_1.5 (e.g. G1743R, D1275N).^52,53^ For mutations belonging to these two categories, we anticipate that *S10s* gene therapy could compensate the reduced *I*_Na_ by interacting with the WT Na_V_1.5, as almost all human patients are heterozygous for their *SCN5A* mutation(s). Since these mutations do not generate functional channels, it is unlikely that *S10s* would interfere other than just augment the WT channel. The fourth category is represented by loss-of-function based on altered gating kinetics (e.g. G514C, I890T),^54,55^ involving activation at more depolarized membrane potentials or inactivation at less depolarized potentials. Most uncertainty remains with regard to potential interaction of *S10s* with these mutated channels. *S10s* gene therapy might be beneficial for treating these mutations but further investigation of the interaction between *S10s* and these mutant channels is needed to better understand the potential therapeutic value in this context.

In addition to the loss-of-function mutations in *SCN5A*, cardiac *I*_Na_ can be reduced due to other causes as well. Genetic variants in non-coding regions have been reported to reduce *SCN5A* expression by altering transcription factor binding sites,^23,56,57^ or by creating microRNA binding sites.^58^ Since these regulations only affect the expression level but not the biophysical properties of Na_V_1.5, we anticipate *S10s* gene therapy could be beneficial for patients carrying such variants and mutations.

Besides the genetic causes, *I*_Na_ reductions can also be caused by remodelling after cardiac injury such as myocardial infarction, contributing to the associated VT/VF.^59^ Locally overexpressed sodium channels and associated conduction restoration could prevent re-entrant arrhythmias in animal models.^4,28^ Our 3D whole-heart *in silico* experiment suggests that application of *S10s* gene therapy eliminates such arrhythmias in an *SCN5A*-haploinsufficient human heart with fibrotic scar tissues. This result further underlines the anticipated applicability of *S10s* gene therapy to restore conduction and prevent malignant arrhythmias. In this context, delivery via direct intramyocardial injections seems particularly appealing, because of the high transduction efficiency that can be achieved with AAV6 gene transfer (as reported in the literature and confirmed by our own observations; AAV6 appears to be the most potent WT serotype, while more potent variants are under development).^60–62^ The anticipated dose needed for injection in a region with slow/abnormal conduction is 1 × 10^12^ VG/injection,^61,63^ which is significantly lower than what is currently applied in cardiac gene therapies that target the heart via the systemic circulation (e.g. 6.7 × 10^13^–1.1 × 10^14^ VG/kg in a recent clinical trial).^64^ Moreover, areas of abnormal conduction are relatively easily identified using electroanatomical mapping, which is common practice in clinical cardiac EP procedures. Functional validation of our proposed approach of direct intramyocardial injections with AAV-S10s gene therapy in porcine models of malignant arrhythmias therefore represents a critical next step before application to human subjects can be considered.^65,66^

The following limitations of our study should be recognized. First, the mechanism of *S10s* function remains incompletely understood. Further in depth biochemical and cell biological studies are needed to reveal details of this mechanism. Second, to test the anti-arrhythmic effect, our *S10s* gene therapy was applied prior to injury in the mouse I/R model, which is different from the anticipated clinical scenario where it will be applied after the disease onset and in a different setting. Third, the relatively small group size in the mouse VT experiment did not have adequate power to detect anything but huge differences. Therefore, the analysis could not rule out the presence of potentially clinically meaningful differences. Fourth, our 3D whole-heart studies could not model cellular heterogeneity in transduction. Yet the finding that *S10s* was still capable in preventing unidirectional block at relatively low transduction efficiencies was reassuring. Moreover, the transduction efficiency with intramyocardial injection of AAV6 is typically very high, i.e. a transduction efficiency of >80%. Last, here we performed *in vivo* testing only in murine models. Further translational testing in large animal models of cardiac arrhythmias represents a high priority to enable clinical trials.

In conclusion, we developed an AAV-based gene therapy utilizing *S10s*, a naturally occurring transcript, for the treatment of sodium current insufficiencies. In mouse and human models, we showed that *S10s* gene therapy increases cardiac CV and prevents cardiac arrhythmias by increasing *I*_Na_ in cardiomyocytes. Our results present *S10s* as a promising gene therapy target for treating cardiac arrhythmias based on conduction abnormalities, and we propose local AAV-mediated gene transfer as the most viable route of administration to treat human subjects.

## Supplementary Material

ehaf053_Supplementary_Data

## Declarations

### Data Availability

The data supporting this study's findings are available from the corresponding authors upon reasonable request.

### Pre-registered Clinical Trial Number

None supplied.
