# Supplementary material for: SCN10A-short gene therapy to restore conduction and protect against malignant cardiac arrhythmias
Source: Eur Heart J. 2025 Feb 20;46(18):1747–62. doi: 10.1093/eurheartj/ehaf053 (PMC12055233; doi:10.1093/eurheartj/ehaf053)
Supplement: ehaf053_Supplementary_Data [file ehaf053_supplementary_data.docx]

# **Supplementary figures**


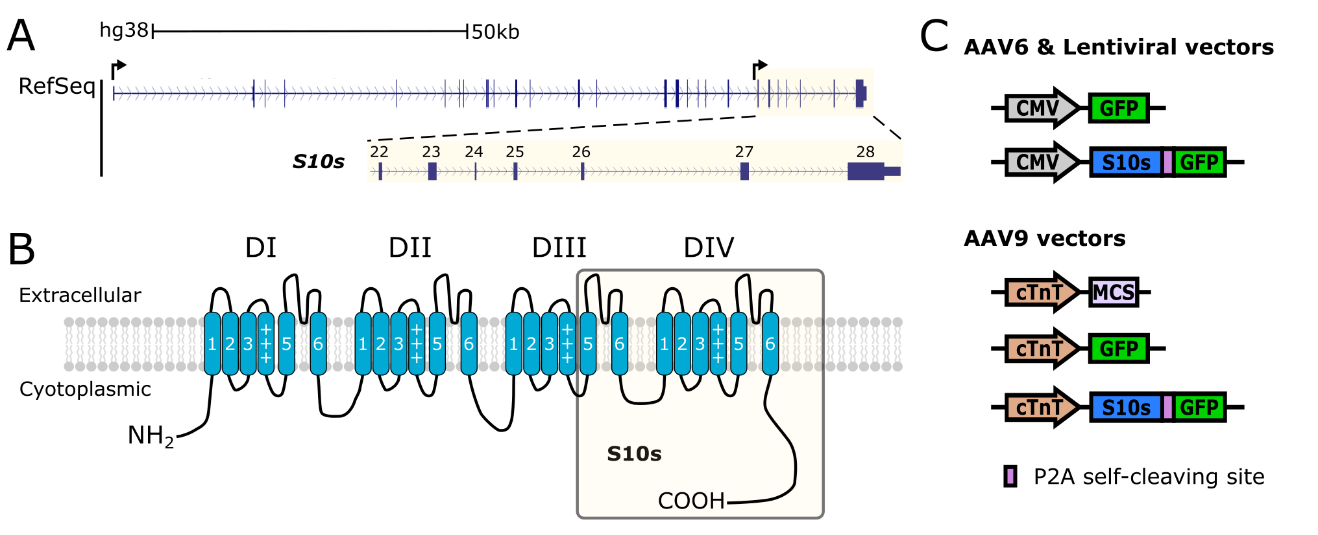
 **Figure S1. Schematic diagrams of *SCN10A-short (S10s)*, its predicted coding product SCN10A-short (S10s), and the viral vectors used in this study.**

(**A**) A UCSC genome browser view of the human *SCN10A* locus and a zoom-in view of the *S10s* region. (**B**) A schematic representation of S10s, the predicted coding product of *S10s*. It contains the C-terminal portion of the full SCN10A channel, including part of domain III, the entire domain IV, and the cytosolic C-terminus. Adapted from Man et al.^1^ (**C**) Viral vectors used in this study. The S10s vectors contain a bicistronic expression cassette including a self-cleaving P2A-GFP. GFP vectors contain only GFP. The MCS vector contains no coding sequence.


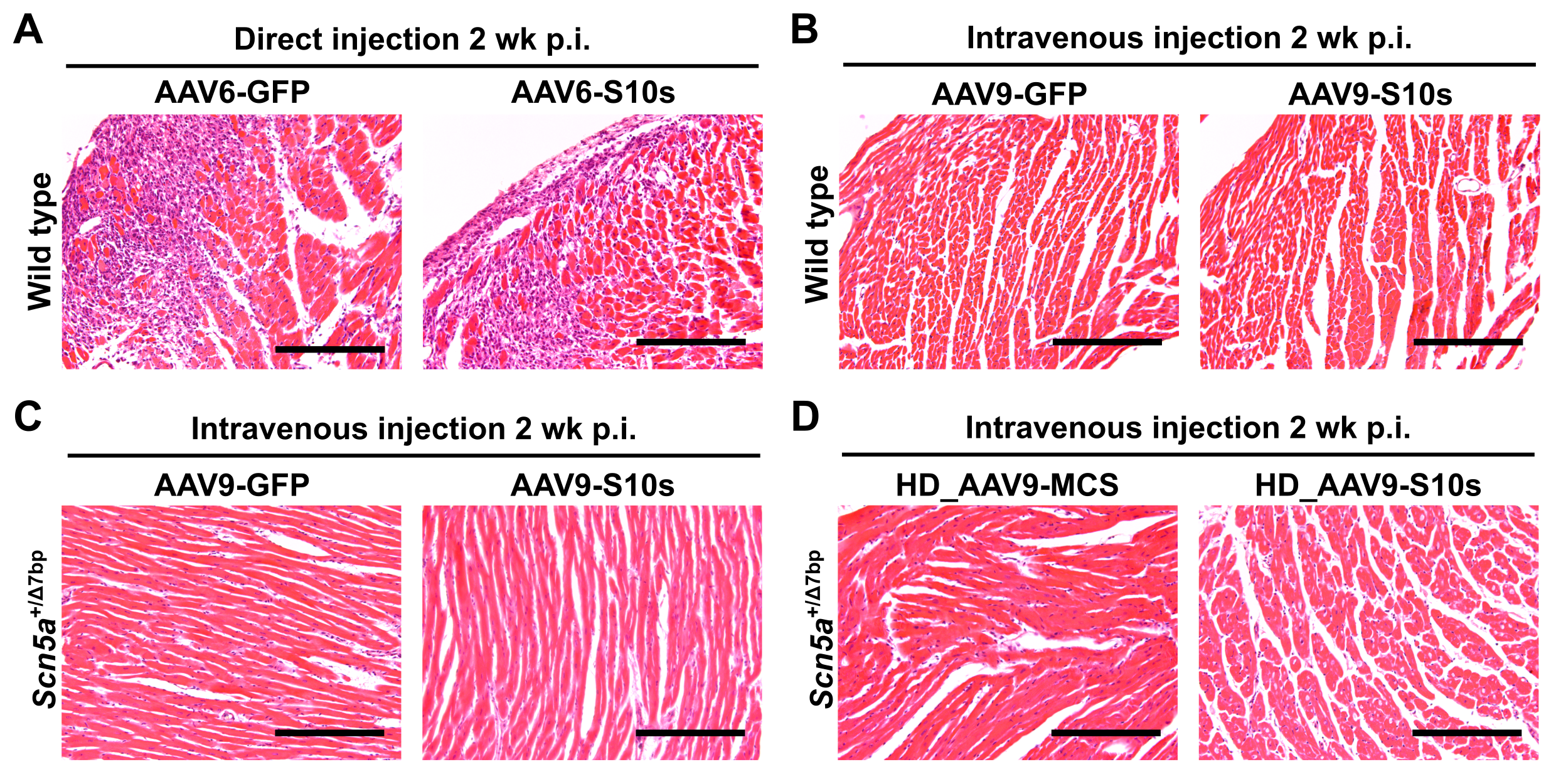


**Figure S2. Hematoxylin and eosin (H & E) staining images of hearts from mice injected with AAV vectors.**

(**A**) Wild type mice following direct intramyocardial injections. (**B**) Wild type mice following intravenous injections. (**C**) *Scn5a^+/Δ7bp^* mice following intravenous injections. (**D**) *Scn5a^+/Δ7bp^* mice following intravenous injections at high dose. Scale bars represent 200 µm. wk p.i., weeks post injection.

**Figure S3. Kinetic parameters of the sodium current in ventricular cardiomyocytes isolated from mice injected with AAV6-GFP or AAV6-S10s.**


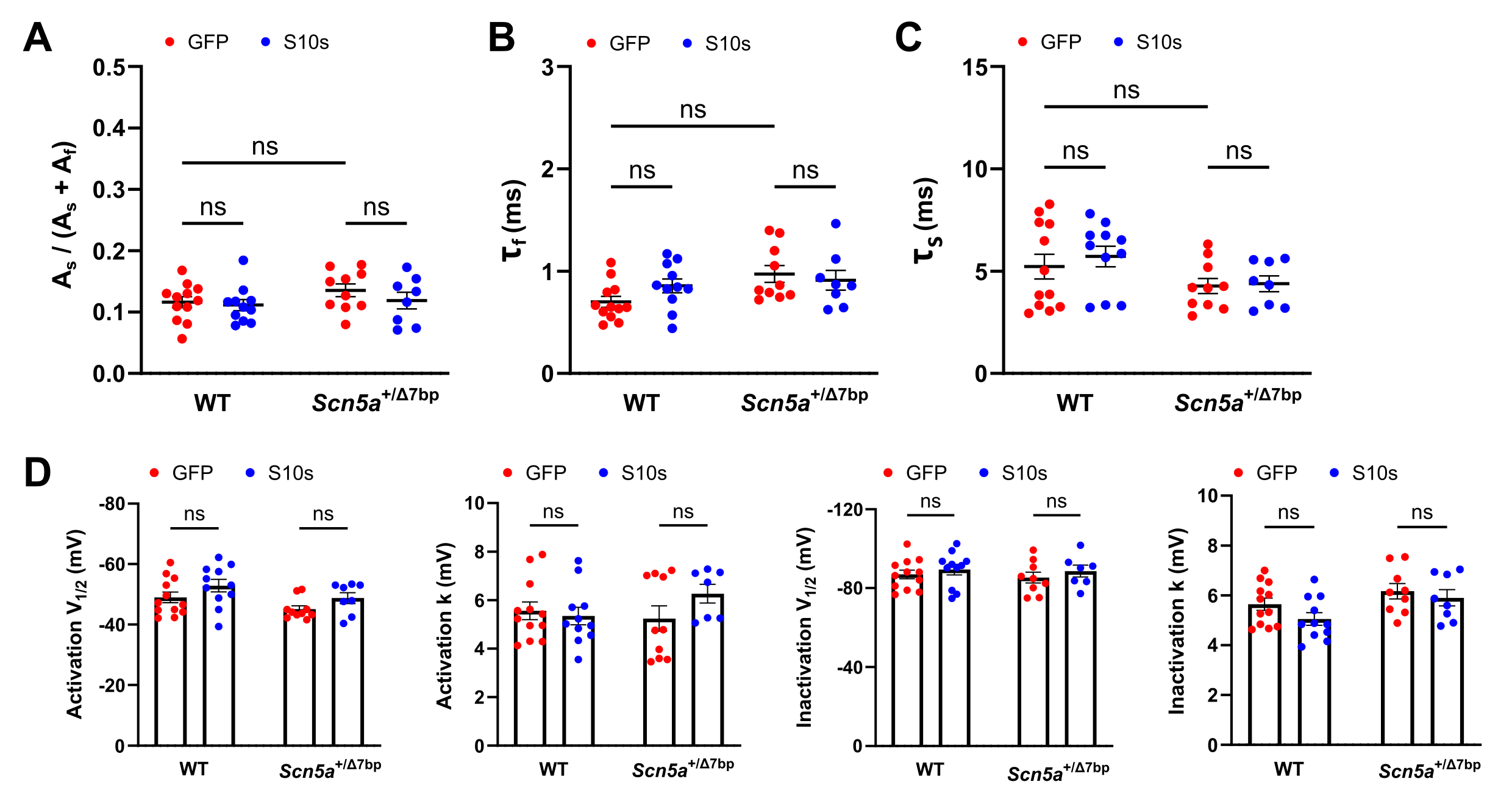


(**A**) Amplitude ratio of the slow inactivation component. (**B**) Time constant of the fast inactivation component. (**C**) Time constant of the slow inactivation component. (**D**) Voltage of half-maximal (in)activation (V_1/2_) and (in)activation slope factor (k) of the sodium current (*I*_Na_) in cardiomyocytes isolated from mice transduced with AAV6-GFP or AAV6-S10s. Data are presented as mean ± SEM. Data were compared using two-way ANOVA with *post-hoc* Fisher’s LSD test. ns, not significant.


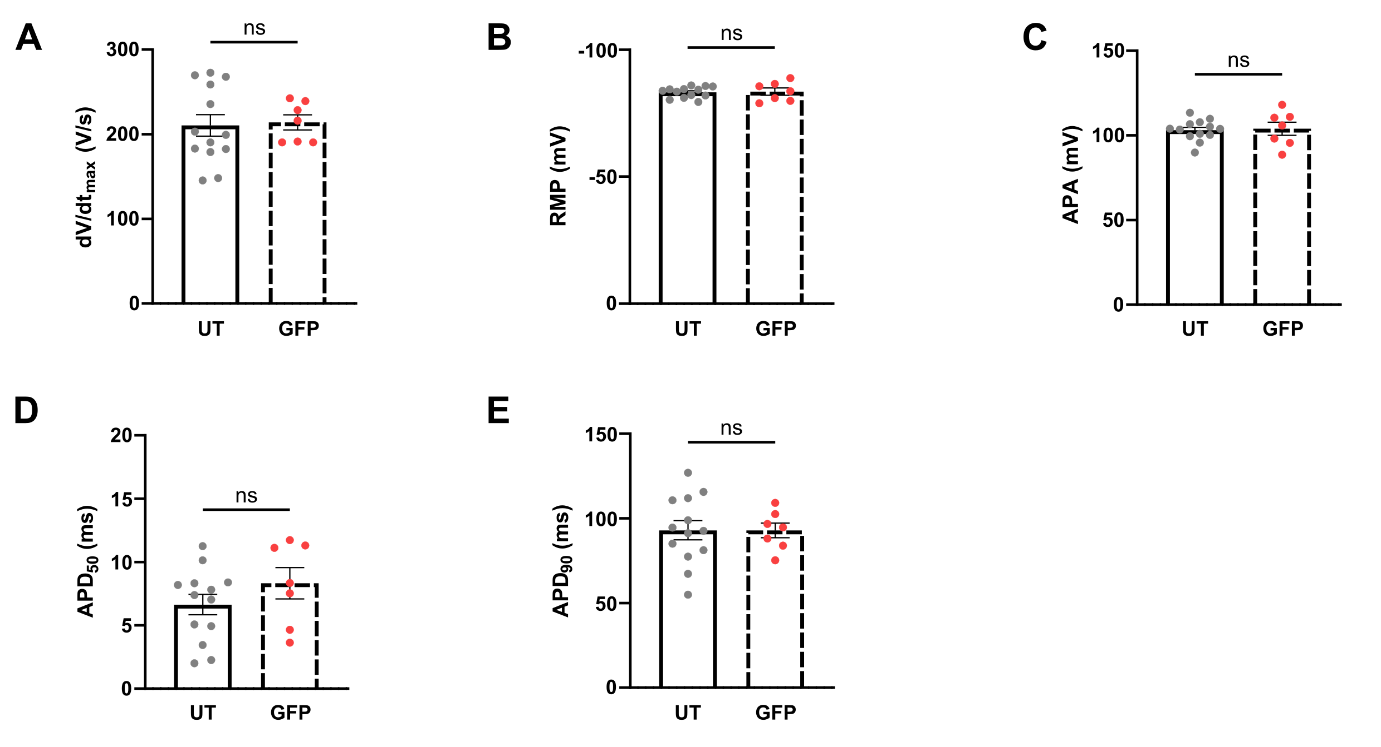


**Figure S4. AAV injection does not affect action potential parameters of mouse cardiomyocytes.**

(**A–E**) Action potential (AP) parameters of cardiomyocytes isolated from untreated mice (UT) or mice injected with AAV6-GFP (GFP). (**A**) Maximal AP upstroke velocity (dV/dt_max_). (**B**) Resting membrane potential (RMP). (**C**) AP amplitude (APA). (**D**) AP duration at 50% of repolarization (APD_50_). (**E**) AP duration at 90% of repolarization (APD_90_). Rightmost bars are taken from Figure 2 and shown for comparison here. Data are presented as mean ± SEM. Data were compared using Student’s t-test. ns, not significant.

**
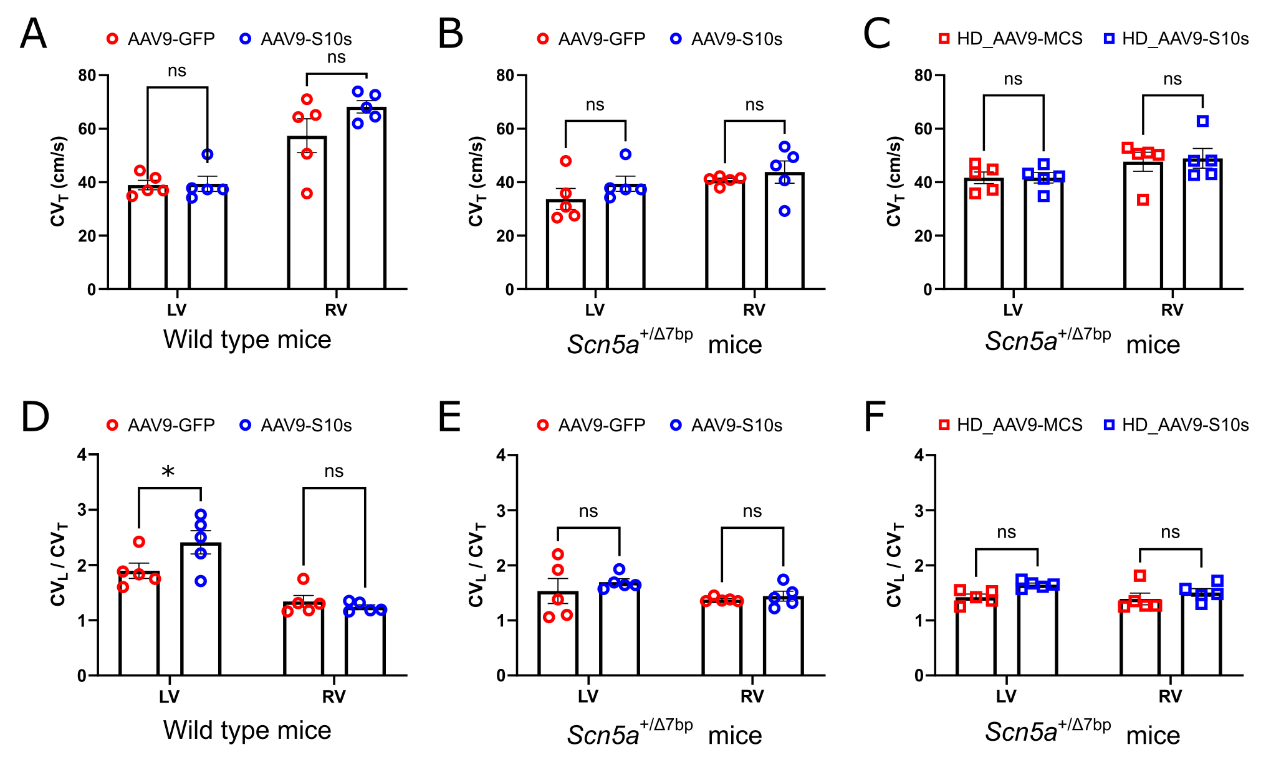
**

**Figure S5. *S10s* gene therapy does not importantly affect the degree of anisotropic conduction.**

(**A-C**) Epicardial transversal conduction velocity (CV_T_) from ventricles of mice injected with AAV vectors. (**A**) Wild type mice injected with AAV9 or AAV9-S10s. (**B**) *Scn5a^+/Δ7bp^* mice injected with AAV9-GFP or AAV9-S10s. (**C**) *Scn5a^+/Δ7bp^* mice injected with HD_AAV9-MCS or HD_AAV9-S10s. (**D-F**) Conduction velocity ratios in mice injected with AAV vectors. (**D**) Wild type mice injected with AAV9 or AAV9-S10s. (**E**) *Scn5a^+/Δ7bp^* mice injected with AAV9-GFP or AAV9-S10s. (**F**) *Scn5a^+/Δ7bp^* mice injected with HD_AAV9-MCS or HD_AAV9-S10s. Data are presented as mean ± SEM. Data were compared using two-way ANOVA with *post-hoc* Fisher’s LSD test. *p < 0.05; ns, not significant.

**
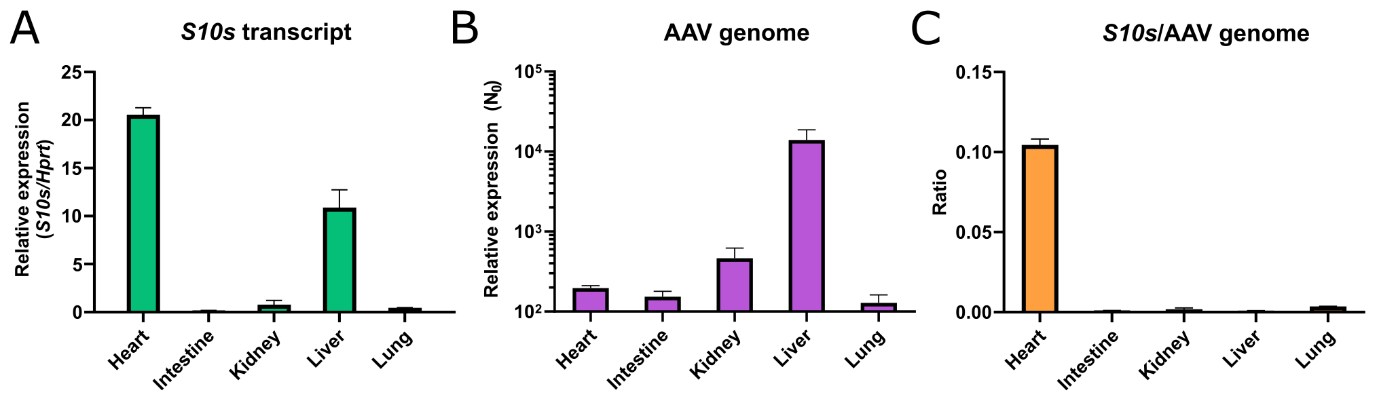
 Figure S6. The bio-distribution of *S10s* transcripts and AAV genomes in *Scn5a^+/Δ7bp^* mice injected with HD_AAV9-S10s (n = 5-6).**

(**A**) *S10s* transcript bio-distribution in *Scn5a*^+/Δ7bp^ mice injected with HD_AAV9-S10s. (**B**) AAV genome bio-distribution in *Scn5a*^+/Δ7bp^ mice injected with HD_AAV9-S10s. (**C**) Ratio between *S10s* transcripts and AAV genomes. Data are presented as mean + SEM.

**
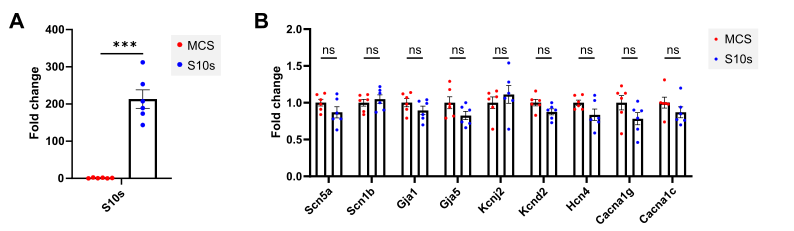
**

**Figure S7. Gene expression changes 4 weeks post injection of HD_AAV9-S10s (n = 6).**

(**A**) Fold change expression of *S10s*. (**B**) Fold change expression of action potential-related ion channel and conduction-related connexin genes. Data are presented as mean ± SEM. Data were compared using Student’s t-test. ns, not significant.

**
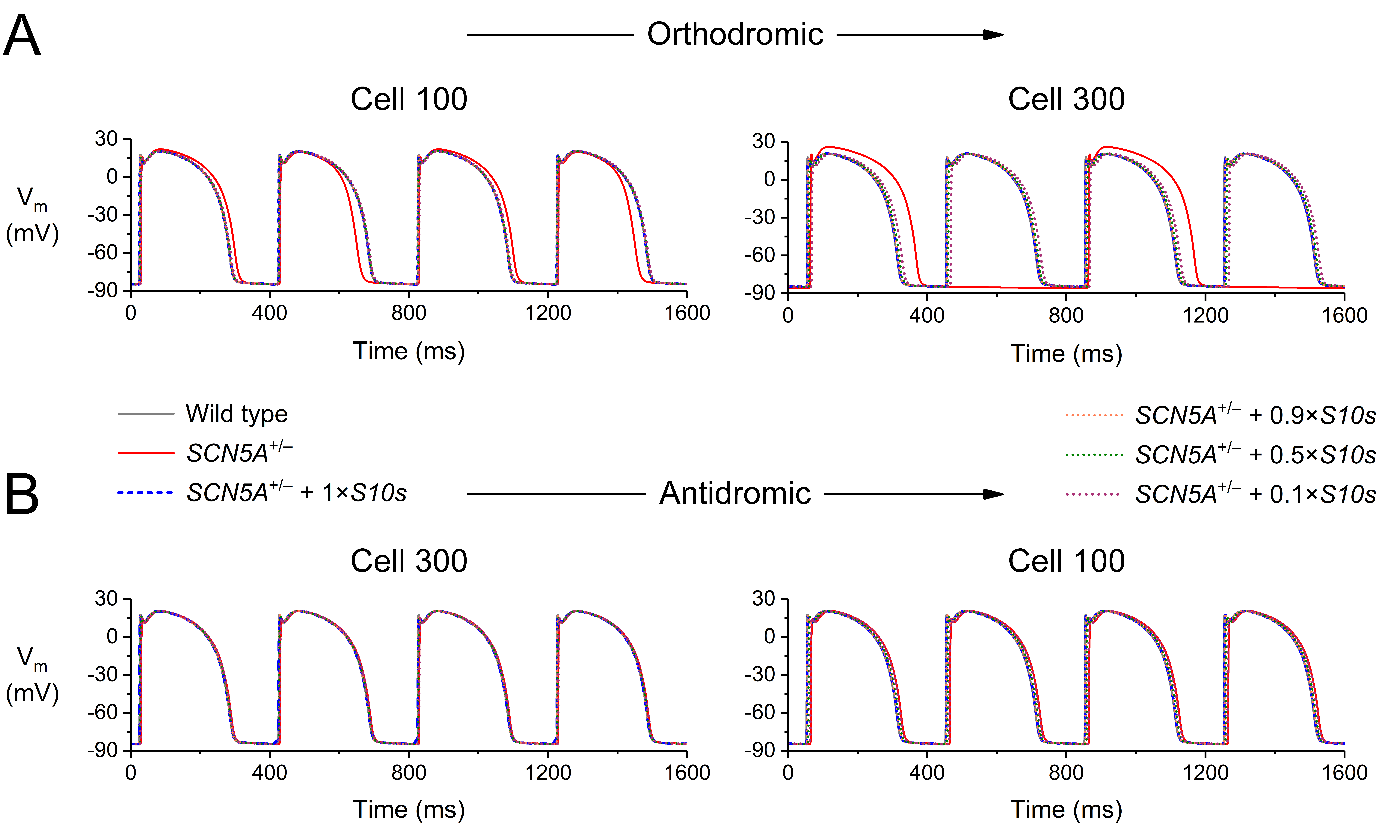
**

**Figure S8. Comparison of orthodromic and antidromic AP conduction in the branched strand of Figure 7C at a stimulus rate of 150 beats/min.**

(**A**) Orthodromic AP conduction. APs of cells #100 (left) and #300 (right) of the 400-cell strand under control conditions (wild type, solid grey line) and in case of *SCN5A*^+/−^ (solid red line) and *SCN5A*^+/−^ + 1×*S10s* (at a transduction efficiency of 100%; dashed blue line). APs at 0.9×, 0.5×, and 0.1× *S10s* overexpression levels are shown as orange, green, and purple dotted lines, respectively. (**B**) Antidromic AP conduction. APs of cells #300 (left) and #100 (right).


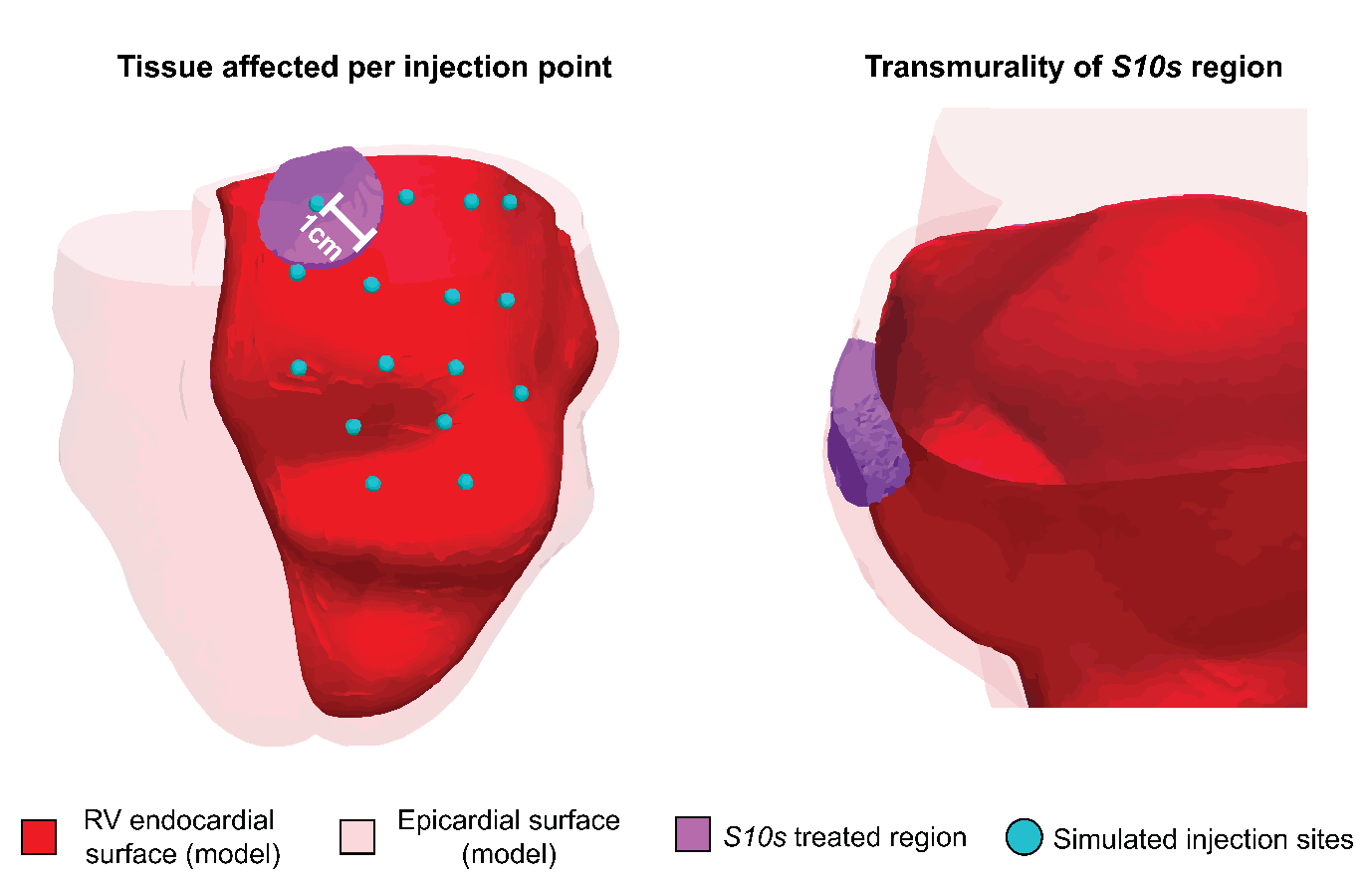


**Figure S9. Spatial distribution of simulated injection sites in a 3D heart model.** *Left:* Sixteen injection points (blue) are displayed on the RV epicardial surface of the heart model (light pink), covering the region associated with the VT circuit shown in Figure 9. The RV endocardial surface is depicted in red. A representative transduced region (purple) from one of the injection points is shown. Each injection point affects a semi-spherical region with a 1 cm radius. Tissue within this region, represented by vertices of the finite-element model, is defined as the *S10s*-transduced area. *Right:* A top-down view demonstrates that each digital injection is transmural, impacting the full thickness of the RV.

# **Supplementary table**

**Table S1. Heart weight and blood chemistry parameters**

**Materials and methods**

**Animals**

Animal care and experiments conform to Directive 2010/63/EU. All animal work was approved by the Animal Ethics Committee of the Academic Medical Center, Amsterdam, and was performed in compliance with the Dutch government guidelines. For cardiomyocytes isolation and conduction velocity measurement, 6-week old male wild type and *Scn5a*^+/∆7bp^ mice on FVB/N background were used. For the ischaemia-reperfusion experiments, 8-week old male C57BL/6Nj mice were used.

**Vector production**

AAV6 vectors were produced by double transfection of HEK293T cells and AAV9 vectors by triple transfections. Low passage HEK293T cells were plated in thirty 145 mm dishes at the density of 1.5 × 10^7^ cells per dish in DMEM-GlutaMax (ThermoFisher Scientific) containing 10% foetal bovine serum (FBS) (Sigma) and 1% penicillin-streptomycin (P/S) (ThermoFisher Scientific). For AAV6 vectors, cells were transfected next day with 33 µg pDP6 and 16 µg AAV transfer plasmids per dish using linear polyethylenimine (PEI) (Polysciences Inc). For AAV9 vectors, cells were transfected with 13 µg Rep2Cap9 plasmid, 21 µg helper plasmid, and 13 µg AAV transfer plasmid per dish. Medium was also replaced during transfection to DMEM containing 1% P/S. Three days after transfection, cells were collected by centrifugation and the medium was concentrated by tangential flow filtration using the ÄKTA flux s system (GE Healthcare) to a final volume of 20 mL. Cells and medium were then combined, frozen and thawed twice followed by DNAseI, Rnase A, and Benzonase treatment. AAV vectors were purified by iodixanol density-gradient ultracentrifugation overnight. The AAV-containing fraction was then collected and concentrated to 1 mL by buffer exchange to PBS containing 0.001% Pluronic F68 using Amicon Ultra-15 100 kDa centrifugal filter units (Millipore). Concentrated AAV vectors were aliquoted and stored at -80°C until use. Genomic titre was determined by qPCR.

Lentiviral vectors were produced by quadruple transfection of HEK293T cells. Cells were plated and cultured as described above. One day after plating, cells were transfected with 3.2 µg envelope plasmid, 2.2 µg RSV-REV plasmid, 5.8 µg MDLG plasmid, and 8.8 µg transfer lentivirus plasmid per dish using linear PEI. Next day medium was changed to DMEM-GlutaMax without FBS. Three days after transfection, supernatant was collected and concentrated to 500 µL by buffer exchange to PBS using Amicon Ultra-15 100 kDa centrifugal filter units (Millipore). Genomic titre was determined using LV900 titration kit (Applied Biological Materials).

**Lentiviral transduction of hiPSC-CMs**

hiPSC-CMs were transduced with lentiviral vectors in culture medium at a multiplicity of infection (MOI) of 10 for 24 hours. The medium was then refreshed every other day until being used in the patch-clamp experiments.

**Whole-cell patch-clamp recordings**

***Cell preparation and data acquisition.*** Left ventricular cardiomyocytes of mice were isolated by an enzymatic dissociation procedure^1^ and ventricular-like hiPSC-CMs were differentiated and dissociated as described previously^2^ from the control hiPSC line LUMC0099iCTRL04. The cell line is registered in the Human Pluripotent Stem Cell Registry (https://hpscreg.eu/cell-line/LUMCi004-A). Single mouse cells were stored at room temperature for at least 45 min in a modified Tyrode’s solution containing (in mmol/L): NaCl 140, KCl 5.4, CaCl_2_ 1.8, MgCl_2_ 1.0, glucose 5.5, HEPES 5.0; pH 7.4 (NaOH). Cells were put into a recording chamber on the stage of an inverted microscope (Nikon Diaphot). For electrophysiological measurements, we selected GFP-positive quiescent single mouse cells with smooth surfaces and single regularly beating hiPSC-CMs. *I*_Na_ and APs were recorded with ruptured and amphotericin-perforated patch-clamp technique, respectively, using an Axopatch 200B amplifier (Molecular Devices, Sunnyvale, CA, USA). Voltage control, data acquisition, and analysis were realized with custom software. Signals were low-pass-filtered with a cutoff of 5 kHz and digitized at 20 and 40 kHz for *I*_Na_ and APs, respectively. Potentials were corrected for the calculated liquid junction potential.^3^ Cell membrane capacitance (C_m_) was estimated by dividing the time constant of the decay of the capacitive transient in response to 5 mV hyperpolarizing voltage clamp steps from -40 mV by the series resistance. For *I*_Na_ measurements, C_m_ and series resistance were compensated by at least 80%.

***I_Na_ measurements*.** *I*_Na_ *was* characterized at room temperature using a bath solution containing (in mmol/L): NaCl 7, CsCl 133, CaCl_2_ 1.8, MgCl_2_ 1.2, glucose 11.0, HEPES 5.0, and pH 7.4 (CsOH). Nifedipine (5 µmol/L) was added to block the L-type calcium current. Pipettes were filled with solution containing (in mmol/L): NaCl 3, CsCl 133, MgCl_2_ 2.0, Na_2_ATP 2.0, TEA-Cl 2.0, EGTA 10.0, HEPES 5.0, and pH 7.2 (CsOH). *I*_Na_ amplitudes and voltage dependency of *I*_Na_ activation were measured by 50 ms depolarizing voltage clamp steps from a holding potential of -120 mV. A two-step protocol consisting of series of 500 ms pulses between -140 and 40 mV from a holding potential of -120 mV, followed by a second 50 ms step to -20 mV, was used to establish the voltage dependency of *I*_Na_ inactivation. Cycle lengths of the protocols were 5 s. *I*_Na_ was defined as the difference between peak current and steady-state current and *I*_Na_ density was calculated by dividing current amplitudes by C_m_. Voltage dependence of activation and inactivation were determined by fitting a Boltzmann function (y=[1+exp{(V-V_1/2_)/k}]^-1^) to the (in)activation data of each individual cell, where V_1/2_ is the voltage of half-maximal (in)activation and k the slope factor (in mV). The time course of current inactivation at -20 mV was fitted by a double-exponential equation: I/I_max_=A_f_ ×exp(-t/τ_f_)+A_s_×exp(-t/τ_s_), where A_f_ and A_s_ are the fractions of the fast and slow inactivation components, and τ_f_ and τ_s_ are the time constants of the fast and slow inactivating components, respectively.

***AP measurements*.** APs were recorded at 36 ± 0.2°C using the modified Tyrode’s solution as bath solution. Pipette solution contained (in mmol/L): K-gluconate 125, KCl 20, NaCl 5.0, amphotericin-B 0.44, HEPES 10, and pH 7.2 (KOH). APs in hiPSC-CMs were measured with dynamic clamp^4^ to inject an in silico *I*_K1_ with a current-voltage relationship resembling Kir2.1 channels with a 2 pA/pF outward peak^5^, resulting in quiescent hiPSC-CMs with an RMP of −80 mV or more negative. APs were elicited at 6 and 1 Hz for murine cardiomyocytes and hiPSC-CMs, respectively, by 3-ms, ≈10-30% suprathreshold current pulses through the patch pipette. AP parameters of individual cells are the average from 10 consecutive APs.

**Intramyocardial injection of AAV**

Animals were injected subcutaneously with buprenorphine (0.075 mg/kg) and carprofen (0.05 mg/kg) for analgesia, at least 30 min prior to surgery. Anaesthesia was induced with 4% isoflurane in 1 L/min O_2_. Mice were shaved, intubated and placed on a heating mat to maintain body temperature. Subsequently, an analgesic mixture consisting of lidocaine (2 mg/kg) and bupivacaine (3 mg/kg) was applied subcutaneously at the site of the incision. Anaesthesia was maintained using ventilation with 2% isoflurane in 1 L/min O_2_. Left thoracotomy was performed at the fourth intercostal space to expose the apex of the heart. To inject the viral vector into the apex a 10 µL Hamilton syringe fitted with a 31-G needle (13 mm, point style 4) was inserted from the anterior LV towards the apex. Five µL of the viral vector solution was slowly administered at each injection site to administer a total volume of 20 µL viral vectors. The thoracotomy and skin were closed with a C-1 12 mm cutting needle with a 6-0 silicone coated braided silk wire (Sofsilk, Covidien). Post-surgery analgesia consisted of 4 days of *ad libitum* carprofen (Rimadyl Cattle, 0.06 mg/ml) in drinking water and wet food.

**Intravenous injection of AAV**

Animals were anesthetized using 4% isoflurane using an induction chamber. The animal was taken out of the chamber and restrained using the thumb and middle finger. Using the index finger skin above the eye is pulled back until the eye slightly protrudes. A 0.3 mL (30G) x 8 mm U-100 insulin needle (BD Micro-Fine) was inserted at an angle of 45° starting around the medial canthus towards the retro-orbital sinus. The construct was slowly injected in one smooth motion in a maximum volume of 100 µL after which the animal was placed on a heating pad until it fully regained consciousness. Mice were monitored 2 days post injection for any abnormalities.

**Mouse electrocardiograms**

Animals were anesthetized by inhaling 4% isoflurane and maintained in anaesthesia with 2% isoflurane in 1 L/min O_2_. Subcutaneous recording electrodes were placed at the left armpit, right armpit and left groin and ECGs were recorded for a period of 1 min. ECG parameters (RR, PR, QRS, QT, and QTc intervals) were calculated from lead II using LabChart Pro 8 (ADInstruments).

**Ischaemia reperfusion**

Animals were injected subcutaneously with buprenorphine (0.075 mg/kg) for analgesia, at least 30 min prior to surgery. Anaesthesia was induced with 4% isoflurane in 1 L/min O_2_. Mice were shaved, intubated and placed on a heating mat to maintain body temperature. Anaesthesia was maintained using ventilation with 2% isoflurane in 1 L/min O_2_. Left thoracotomy was performed at the third intercostal space to expose the hearts superior left ventricular wall and left atrium. A small piece of sterile gaze soaked in physiological saline solution was used to push the left atrium away. The left anterior descending coronary artery (LAD) was identified and a 6.5 mm 3/8c taper point 8-0 polyamide 6/6 monofil suture (Ethilon, Ethicon) was placed around the LAD proximal to its branches. A cut piece of a 22G intubation cannula was placed on top of the occlusion location and the ligation was made by tying a double surgeons knot on top of it. Myocardial ischaemia was confirmed by regional cyanosis and S elevation from ECG. Five minutes later, the artery was reperfused by pulling the cannula tubing with fine tipped forceps. Reperfusion was confirmed by a rapid colour change in the surface of the myocardium. Ventricular tachycardia (VT) was defined as 4 or more consecutive ectopic ventricular beats. When VT was observed, its incidence, rate, and duration were analysed.

**Immunofluorescence staining**

Cells were fixed in 4% paraformaldehyde (PFA) for 10 min at room temperature. Hearts were fixed in 4% PFA overnight and sectioned at 7 µm. Sections were deparaffinised and dehydrated by a series of ascending ethanol concentrations. For antigen retrieval, sections were boiled in unmasking solution (H3300, Vector). Cells and sections were blocked in 4% bovine serum albumin and incubated with chicken anti-GFP (1:500, Aves Labs, GFP-1020) and mouse anti-2A (1:1000, Novus Biologicals, NBP2-59627). DAPI (1 µg/mL, Sigma, D 9542) was used as a nuclear stain. Fluorescence images were acquired using a Leica DM6000 fluorescence microscope or a Leica TCS SP8 confocal microscope (Leica Microsystems).

**Hematoxylin and eosin staining**

Sections were deparaffinised, dehydrated by a series of ascending ethanol concentrations, and stained with hematoxylin and eosin. Images were acquired using a Leica DM5000 fluorescence microscope (Leica Microsystems).

**RNA isolation and RT-qPCR**

Total RNA was isolated from cardiac tissue using NucleoSpin RNA (Macherey-Nagel) according to the manufacturer’s protocol. cDNA library was transcribed from 500-1000 ng total RNA with oligo-dT primers (125 µmol/L) and the Superscript II system (Invitrogen). Quantitative PCR was performed using the LightCycler 480 Real-Time PCR system (Roche). Relative start concentration was calculated using LinRegPCR^6^ and values were normalized to *Hprt* expression level.

**Optical mapping of APs in mouse hearts**

The mice were killed by cervical dislocation after which the heart was excised, cannulated and mounted on a Langendorff perfusion set-up. The hearts were perfused with Tyrode’s solution (37°C) containing (in mmol/L): NaCl 128, KCl 4.7, CaCl_2_ 1.45, MgCl_2_ 0.6, NaHCO_3_ 27, NaH_2_PO_4_ 0.4, and glucose 11. The solution was maintained at 7.4 pH by equilibration with a mixture of 95% O_2_ and 5% CO_2_. Hearts were incubated with 15 µmol/L di-4-ANEPPS (Bio-Techne) in 10 mL Tyrode’s solution after which they were placed in the optical mapping setup and perfused with 10 µmol/L blebbistatin (Bio-Techne) in Tyrode’s solution to reduce motion artefacts. Excitation light was provided by a 5-watt power LED (filtered 510 ± 35 nm). Fluorescence (filtered > 610 nm) was transmitted through a tandem lens system on a CMOS sensor (100 x 100 elements, sampling rate 5 kHz, MiCAM Ultima, SciMedia). Activation patterns were measured during epicardial stimulation at an interval of 120 ms. Optical APs and conduction velocity were analysed with custom software.

**Computer simulations of linear strands of ventricular myocytes**

The functional effect of a complete loss-of-function mutation in *SCN5A* and the conduction improving effect of *S10s* at different levels of transduction efficiency and *S10s* overexpression were assessed by computer simulations of a linear strand of cardiomyocytes, using the Ten Tusscher et al. human left ventricular cell model,^7^ as updated by Ten Tusscher and Panfilov,^8^ to describe individual cells. When simulating AP propagation in strands of cells, the myoplasmic resistivity was set to 150 Ω∙cm^9^ and the conduction velocity was computed across the middle third of the strand. In initial simulations, we tested whether the number of 400 cells in the branched and non-branched strands was large enough to rule out any effects of the ‘sealed ends’ at both ends of the strand. We obtained indistinguishable results with strands of 200–800 cells and decided to use the number of 400 cells in our final experiments in order to balance numerical accuracy and computational cost. Software was compiled as a 32-bit Windows application using Intel Visual Fortran Composer XE 2013 and run on an Intel Core i7 processor-based workstation. For the numerical integration of differential equations we applied a simple and efficient Euler-type integration scheme with a 1 µs time step.^10^ All simulations were run for a period of 50 stimuli and the results shown are from the final 1–4 stimuli. In initial simulations, we tested whether the number of 50 stimuli was large enough to reach steady state behaviour by comparing the simulation results obtained with 20–100 stimuli. When superimposed, simulation results obtained with 50–100 stimuli were indistinguishable.

**Computer simulations using 3D human ventricular model**

We reconstructed the biventricular heart model from late-gadolinium cardiac magnetic resonance images. In line with previous studies modelling genetic heart diseases,^11^ we categorized the myocardium of the model into three distinct tissue types: non-fibrotic, diffuse fibrotic and dense scar tissue. Subsequently, we created tetrahedral meshes for finite-element EP simulations (Mimics Innovation Suite; Materialise, Leuven, Belgium), and assigned fibre orientation to each element of the model to ensure realistic electrical conduction patterns using a validated rule-based method^12^.

Aiming to demonstrate the capability of *S10s* gene therapy in reducing arrhythmogenic propensity, we first created a baseline human whole-heart model with a loss-of-function *SCN5A* mutation. For this purpose, we developed a new *SCN5A^+/−^* cell model, incorporating EP remodelling associated with this mutation by modifying the Ten Tusscher and Panfilov human ventricular cell model with a 50% decrease in maximal sodium channel conductance. This *SCN5A^+/−^* cell model was applied to all non-fibrotic regions of the heart model. For cardiomyocytes in fibrotic regions, we incorporated additional modifications based on experimental data, which have been validated in previous studies.^13,14^ Scar tissue was modelled as non-conductive regions.

To describe the application of *S10s* therapy, we further modified the *SCN5A*^+/-^ cell model with EP properties based on the experimental findings of this study. This included increasing the residual maximal sodium current by 80.4%, resulting in an *SCN5A*^+/-^ + *S10s* model with a sodium current at 90.2% of the baseline Ten Tusscher and Panfilov cell model.

To simulate the electrical activity of the heart, we used the openCARP software package.^15^ Full details on the simulation can be found in previous publications.^11,16^ The model was paced sequentially from nine uniformly distributed endocardial RV locations, spanning from base to apex, using a validated rapid pacing protocol that replicates clinical electrophysiology study conditions.^17^ After inducing the re-entrant circuits, we analysed the activation maps to determine the location and morphology of the VT circuit.

We then digitally applied *S10s* gene therapy to target the VT circuit intended for termination. To simulate the *S10s* injection, we selected 16 points on the RV of the heart model to fully cover the region associated with the VT circuit (Figure 9E). Details of the spatial distribution of simulated injection sites have been described in Figure S9. Each injection point was assumed to affect the cardiac tissue within a 1cm radius semi-sphere (Figure S9, left). This setup created a fully transmural *S10s*-transduced region due to the thin RV wall (Figure S9, right). All finite elements within this area, defined by these 16 points, were assigned the EP properties of the *SCN5A*^+/-^ + *S10s* cell model. The success of this experiment was demonstrated by the non-inducibility of VT in the *SCN5A*^+/-^ heart model following localized *S10s* therapy.

**Statistical analysis**

GraphPad Prism version 9 software (GraphPad Software Inc.) was used for statistical analysis. Numerical data are presented as mean ± standard error of the mean (SEM) of at least three biological replicates. Two independent groups were compared by unpaired Student’s t-test and multiple independent groups were compared by analysis of variance (ANOVA) followed by the *post-hoc* Fisher’s LSD test for multiple comparisons. Categorical data were presented as absolute values and percentages. Categorical data were compared by Fisher’s exact test. All tests were performed two-sided and statistical significance was considered for p < 0.05.

# **References**

1. Man JCK, Bosada FM, Scholman KT, Offerhaus JA, Walsh R, Van Duijvenboden K, et al. Variant intronic enhancer controls *SCN10A-short* expression and heart conduction. *Circulation*. 2021;144:229-242. doi: 10.1161/CIRCULATIONAHA.121.054083

2. Li J, Wiesinger A, Fokkert L, Boukens BJ, Verkerk AO, Christoffels VM, et al. Molecular and electrophysiological evaluation of human cardiomyocyte subtypes to facilitate generation of composite cardiac models. *J Tissue Eng*. 2022;13:20417314221127908. doi: 10.1177/20417314221127908

3. Barry PH, Lynch JW. Liquid junction potentials and small cell effects in patch-clamp analysis. *J Membr Biol*. 1991;121:101-117. doi: 10.1007/BF01870526

4. Wilders R. Dynamic clamp: a powerful tool in cardiac electrophysiology. *J Physiol*. 2006;576:349-359. doi: 10.1113/jphysiol.2006.115840

5. Meijer van Putten RME, Mengarelli I, Guan K, Zegers JG, Van Ginneken ACG, Verkerk AO, et al. Ion channelopathies in human induced pluripotent stem cell derived cardiomyocytes: a dynamic clamp study with virtual *I*_K1_. *Front Physiol*. 2015;6:7. doi: 10.3389/fphys.2015.00007

6. Ruijter JM, Ramakers C, Hoogaars WMH, Karlen Y, Bakker O, Van den Hoff MJB, et al. Amplification efficiency: linking baseline and bias in the analysis of quantitative PCR data. *Nucleic Acids Res*. 2009;37:e45. doi: 10.1093/nar/gkp045

7. Ten Tusscher KHWJ, Noble D, Noble PJ, Panfilov AV. A model for human ventricular tissue. *Am J Physiol Heart Circ Physiol*. 2004;286:H1573-H1589. doi: 10.1152/ajpheart.00794.2003

8. Ten Tusscher KHWJ, Panfilov AV. Cell model for efficient simulation of wave propagation in human ventricular tissue under normal and pathological conditions. *Phys Med Biol.* 2006;51:6141-6156. doi: 10.1088/0031-9155/51/23/014

9. Wilders R. Arrhythmogenic right ventricular cardiomyopathy: considerations from in silico experiments. *Front Physiol*. 2012;3:168. doi: 10.3389/fphys.2012.00168

10. Rush S, Larsen H. A practical algorithm for solving dynamic membrane equations. *IEEE Trans Biomed Eng*. 1978;25:389-392. doi: 10.1109/tbme.1978.326270

11. Zhang Y, Zhang K, Prakosa A, James C, Zimmerman SL, Carrick R, et al. Predicting ventricular tachycardia circuits in patients with arrhythmogenic right ventricular cardiomyopathy using genotype-specific heart digital twins. *Elife*. 2023;12:RP88865. doi: 10.7554/eLife.88865

12. Bayer JD, Blake RC, Plank G, Trayanova NA. A novel rule-based algorithm for assigning myocardial fiber orientation to computational heart models. *Ann Biomed Eng*. 2012;40:2243-2254. doi: 10.1007/s10439-012-0593-5

13. O'Hara RP, Binka E, Prakosa A, Zimmerman SL, Cartoski MJ, Abraham MR, et al. Personalized computational heart models with T1-mapped fibrotic remodeling predict sudden death risk in patients with hypertrophic cardiomyopathy. *Elife*. 2022;11:e73325. doi: 10.7554/eLife.73325

14. Shade JK, Cartoski MJ, Nikolov P, Prakosa A, Doshi A, Binka E, et al. Ventricular arrhythmia risk prediction in repaired Tetralogy of Fallot using personalized computational cardiac models. *Heart Rhythm*. 2020;17:408-414. doi: 10.1016/j.hrthm.2019.10.002

15. Plank G, Loewe A, Neic A, Augustin C, Huang YL, Gsell MAF, et al. The openCARP simulation environment for cardiac electrophysiology. *Comput Methods Programs Biomed*. 2021;208:106223. doi: 10.1016/j.cmpb.2021.106223

16. Prakosa A, Arevalo HJ, Deng D, Boyle PM, Nikolov PP, Ashikaga H, et al. Personalized virtual-heart technology for guiding the ablation of infarct-related ventricular tachycardia. *Nat Biomed Eng*. 2018;2:732-740. doi: 10.1038/s41551-018-0282-2

17. Arevalo HJ, Vadakkumpadan F, Guallar E, Jebb A, Malamas P, Wu KC, et al. Arrhythmia risk stratification of patients after myocardial infarction using personalized heart models. *Nat Commun*. 2016;7:11437. doi: 10.1038/ncomms11437

# **List of abbreviations**

2D/3D Two/three-dimensional

AAV Adeno-associated virus

AAV6/9 Adeno-associated virus serotype 6 or 9

AF Arial fibrillation

AP Action potential

APA Action potential amplitude

APD_50_/APD_90_ Action potential duration at 50% / 90% repolarization

BrS Brugada syndrome

C_m_ Cell membrane capacitance

cTnT Cardiac troponin T

CV Conduction velocity

CV_L_/CV_T_ Longitudinal / transversal conduction velocity

dV/dt_max_ Maximal upstroke velocity

EP Electrophysiology

FBS Foetal bovine serum

g_j_ Gap junctional conductance

H & E staining Hematoxylin and eosin staining

HD High dose

hiPSC-CM Human induced pluripotent stem cell-derived cardiomyocyte

*I*_Na_ Sodium current

LGE-CMR Late-gadolinium enhanced cardiac magnetic resonance

MOI Multiplicity of infection

Na_V_1.5 Cardiac voltage-gated sodium channel α-subunit

P/S Penicillin-streptomycin

PCCD Progressive cardiac conduction disease

PEI Polyethylenimine

RMP Resting membrane potential

RT-qPCR Reverse transcription quantitative PCR

S10s SCN10A-short

*Scn5a^+/∆7bp^* A *Scn5a*-haploinsufficient mouse model

SSS Sick sinus syndrome

VG Viral genome

VT/VF Ventricular tachycardia/fibrillation
